# Isolation and Biological Activity of Iezoside and Iezoside B, SERCA Inhibitors from Floridian Marine Cyanobacteria

**DOI:** 10.3390/md21070378

**Published:** 2023-06-27

**Authors:** Sofia Kokkaliari, Danmeng Luo, Valerie J. Paul, Hendrik Luesch

**Affiliations:** 1 Department of Medicinal Chemistry and Center for Natural Products, Drug Discovery and Development (CNPD3), University of Florida, 1345 Center Drive, Gainesville, FL 32610, USA; 2 Smithsonian Marine Station at Ft. Pierce, 701 Seaway Drive, Fort Pierce, FL 34949, USA

**Keywords:** anticancer, intracellular calcium, marine cyanobacteria, SERCA inhibitor

## Abstract

Marine cyanobacteria are a rich source of bioactive natural products. Here, we report the isolation and structure elucidation of the previously reported iezoside (**1**) and its C-31 O-demethyl analogue, iezoside B (**2**), from a cyanobacterial assemblage collected at Loggerhead Key in the Dry Tortugas, Florida. The two compounds have a unique skeleton comprised of a peptide, a polyketide and a modified sugar unit. The compounds were tested for cytotoxicity and effects on intracellular calcium. Both compounds exhibited cytotoxic activity with an IC_50_ of 1.5 and 3.0 μΜ, respectively, against A549 lung carcinoma epithelial cells and 1.0 and 2.4 μΜ against HeLa cervical cancer cells, respectively. In the same cell lines, compounds **1** and **2** show an increase in cytosolic calcium with approximate EC_50_ values of 0.3 and 0.6 μΜ in A549 cells and 0.1 and 0.5 μΜ, respectively, in HeLa cells, near the IC_50_ for cell viability, suggesting that the increase in cytosolic calcium is functionally related to the cytotoxicity of the compounds and consistent with their activity as SERCA (sarcoplasmic/endoplasmic reticulum Ca^2+^-ATPase) inhibitors. The structure–activity relationship provides evidence that structural changes in the sugar unit may be tolerated, and the activity is tunable. This finding has implications for future analogue synthesis and target interaction studies.

## 1. Introduction

Calcium ions (Ca^2+^) regulate various cellular processes by activating gene transcription, cell proliferation and migration [1,2]. The cytosolic levels of calcium are maintained at sub-micromolar concentrations, while the extracellular levels are at high millimolar concentrations. Most of the intracellular Ca^2+^ is stored in the endoplasmic reticulum (ER), which is enriched in calcium-binding proteins [1]. Calcium is exchanged between the cytosol and the ER in three different ways: channels, which allow transport from the ER to the cytosol; exchangers of Na^+^/Ca^2+^, which use the Na^+^ gradient to transport Ca^2+^; and pumps (sarcoplasmic/endoplasmic reticulum Ca^2+^-ATPase, SERCA), which transport Ca^2+^ against the concentration gradient into the ER [2,3]. In recent years, it has been reported that cancer cells have altered expression of SERCA pumps, making them a potential target. Inhibition of the SERCA pump leads to high levels of Ca^2+^ in the cytosol, which is toxic and, if prolonged, results in growth arrest and apoptosis of the cells [4,5].

Over 30,000 marine natural products have been identified, with many of them demonstrating some type of biological activity and marine cyanobacteria emerging as an important source of drug leads [6,7,8]. To date, there are several natural products that inhibit the different isoforms of SERCA, including thapsigargin, cyclopiazonic acid, biselyngbyasides, kurahyne and iezoside (**1**) [9,10,11,12,13,14,15,16,17]. Thapsigargin, a sesquiterpene lactone isolated from *Thapsia garganica*, is a potent SERCA inhibitor that binds irreversibly and results in cell apoptosis [4,18]. An analogue of thapsigargin, mipsagargin is currently in clinical trials against hepatocellular carcinoma [18,19,20,21]. Biselyngbyasides are a family of cyanobacterial secondary metabolites that inhibit SERCA. The binding site of biselyngbyasides was identified through co-crystallization with the Ca^2+^ pump and was shown to be in a distinct site from that of thapsigargin [22].

**Figure 1:**
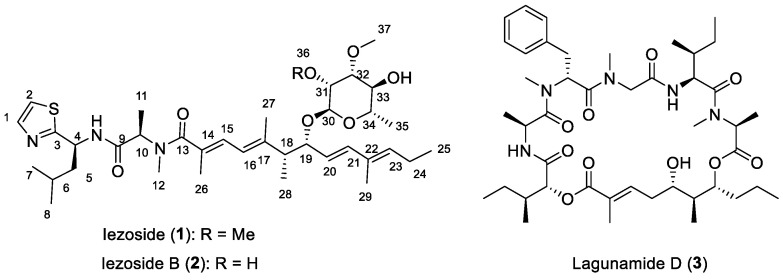
Structures of iezoside (**1**), iezoside B (**2**) and lagunamide D (**3**).

Iezoside (**1**) (Figure 1) was previously isolated from a filamentous marine cyanobacterium collected from Ie Island, Okinawa, Japan, that was identified based on morphology as *Leptochromothrix valpauliae*. Molecular phylogenetic analysis indicated that while the cyanobacterium was related to *Leptochromothrix* spp., it was phylogenetically distinct and likely represented a new species (Figure S1 in [13]). Iezoside consists of three moieties: a polyketide unit, a peptide unit and a rhamnose sugar. The planar structure, double bond geometry and relative configuration of the sugar unit were determined by NMR. The absolute configuration of the molecule was determined by amino acid analysis, Mosher’s method for the sugar moiety and circular dichroism for the polyketide unit. The uncertain C18–C19 configuration required the total synthesis (16 steps LLS, 4.4% total yield) to conclusively assign the complete 3D structure. Using computational methods and electronic circular dichroism (ECD), the compound was limited to two potential isomers, 18*R*,19*R* or 18*S*,19*R*. The total synthesis indicated that **1** had the 18*R*,19*R* configuration. Iezoside (**1**) was identified as a SERCA inhibitor that promotes cell cycle delay and induces apoptosis pathways, with an IC_50_ for cell viability of 6.8 nM against HeLa cells and a K_i_ of 7.1 nM on SERCA1a [13].

We previously reported the discovery of lagunamide D (**3**) (Figure 1), a 26-membered macrocycle from a collection of marine cyanobacteria comprised of mainly *Dichothrix* sp. and *Lyngbya* sp. Compound **3** was shown to be a mitochondrial cytotoxin, with potent antiproliferative activity and an IC_50_ of 7.1 nM against A549 lung adenocarcinoma cells [23,24]. The same extract and fraction that yielded **3** was further investigated based on its NMR profile. Here, we report the isolation of iezoside (**1**) and a new, O-demethyl analogue, iezoside B (**2**) (Figure 1) [13]. Both compounds were isolated in sub-milligram quantities. Iezoside (**1**) was four-fold more abundant than iezoside B (**2**), and logically, we first determined the structure of the major compound, which provided clues about the structural differences of the new minor analogue. Our structure determination of iezoside B (**2**) relied on the NMR comparison in DMSO-*d*_6_, which required us to first conclusively demonstrate that the structures (except the methylation pattern) are identical. Since iezoside (**1**) from Japan was shown to be a cytotoxic and potent SERCA inhibitor, **2** was tested for its cytotoxicity and its effect on intracellular calcium. We compared the potencies of **1** and **2** side-by-side to interrogate the effect of the methylation pattern in the sugar moiety on its activity.

## 2. Results

### 2.1. Isolation and Structure Elucidation

The cyanobacterial assemblage was lyophilized and extracted using 1:1 EtOAc/MeOH followed by 1:1 EtOH:H_2_O and further partitioned between EtOAc and H_2_O, followed by partitioning the polar layer between n-BuOH and H_2_O. The EtOAc soluble portion was further subjected to fractionation using reversed-phase chromatography to yield 0.8 mg of iezoside (**1**) and 0.2 mg of iezoside B (**2**).

**Figure 2:**
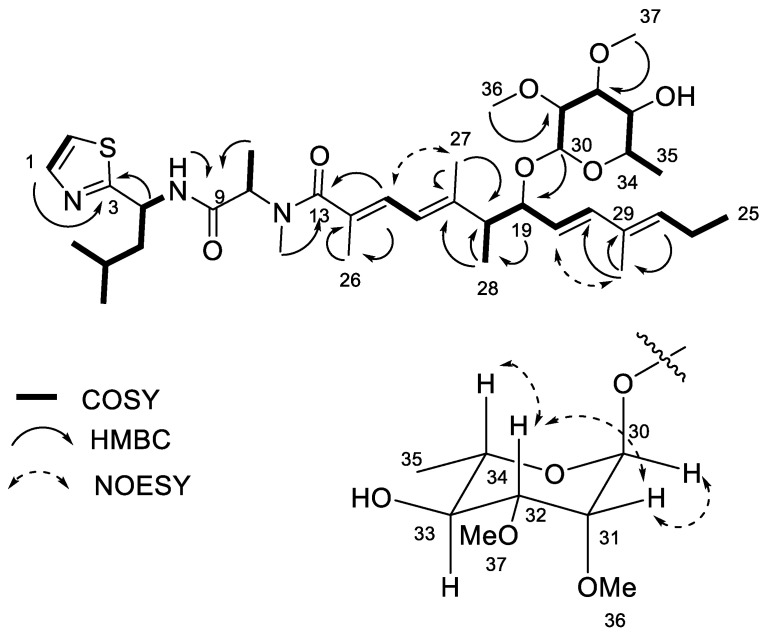
Key COSY, HMBC and NOESY data for iezoside (**1**).

Iezoside (**1**) was isolated as a colorless oil with a molecular formula of C_37_H_59_N_3_O_7_S ([M+H]^+^ 690.4104, calcd. for C_37_H_60_N_3_O_7_S, 690.4152) as determined by HRESIMS. The structure was established in three parts by NMR analysis in DMSO-*d*_6_, while data for iezoside (**1**) from the Okinawan cyanobacterium were published in acetone-*d*_6_ after we had completed our analysis [13]. Briefly, a tripeptide unit was determined by COSY and HMBC correlations as Thz-Leu-*N*-Me-Ala (Table 1, Figure 2). The broadening of the H-10 (δ_H_ 4.83), H-11 (δ_H_ 1.29) and H-12 (δ_H_ 2.78) as well as the lack of HSQC signals were observed in DMSO-*d*_6_, similarly to the published **1** [13]. The broadening was attributed to the slow conformational exchange of the *N*-Me-Ala residue. The polyketide unit was established using the COSY and HMBC correlations in Figure 2 and Table 1, similarly as described [13]. H-21 (δ_H_ 6.14) had to be part of a trans double bond (*J =* 15.7 Hz) with H-20 (δ_H_ 5.29). H-16 (δ_H_ 6.09) was part of a conjugated diene with *s*-trans configuration (*J =* 11.2) with H-15 (δ_H_ 6.32), which also showed HMBC correlation to C-26 (δ_C_ 14.3). To complete the polyketide unit, H-26 (δ_H_ 1.83) showed HMBC correlations to C-14 (δ_C_ 130.2) and C-13 (δ_C_ 173.0). The two units were connected based on the HMBC correlations of H-26 and H-12 (δ_H_ 2.78) to C-13. Finally, the sugar moiety was determined as a 2,3-*O*-dimethyl-methylpentose, based on COSY and HMBC correlations and connected to the rest of the structure based on the HMBC correlations of H-30 (δ_H_ 4.69) to C-19 (δ_C_ 78.2), completing the planar structure.

**Figure 3:**
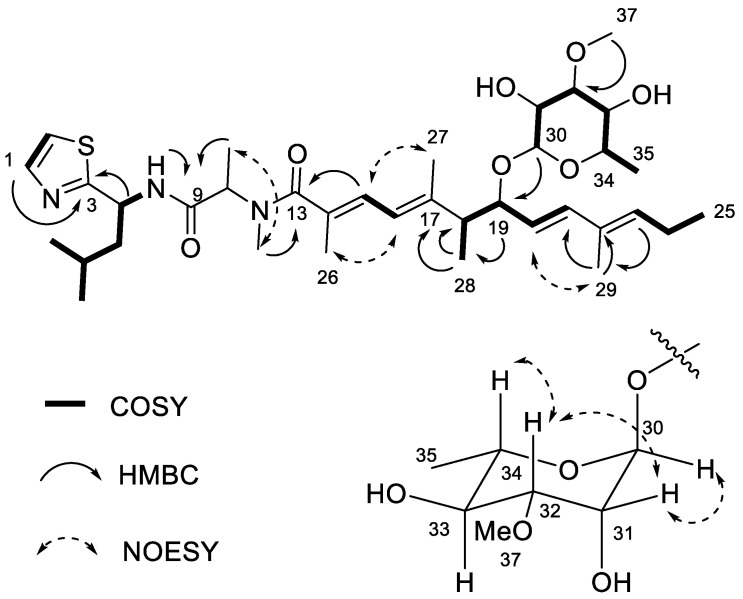
Key COSY, HMBC and NOESY data for iezoside B (**2**).

Iezoside B (**2**) was isolated from the same fraction as iezoside (**1**) and had a molecular formula of C_36_H_57_N_3_O_7_S as determined by HRESIMS ([M+H]^+^ 676.3990 calcd. 676.3954) with 10 degrees of unsaturation. Compound **2** differs by 14 mass units (CH_2_) from **1**, which is attributed to the lack of the methoxy group at C-31 (Figure 1, Figure 2 and Figure 3), as detailed below.

The structure of **2** was established based on 1D and 2D NMR analysis in DMSO-*d*_6_ (Table 1, Figure 3). The thiazole ring was established based on characteristic ^1^H and ^13^C NMR chemical shifts (Table 1), coupled with COSY and HMBC data. H-1 (δ_H_ 7.70) showed a COSY correlation to H-2 (δ_H_ 7.58) and an HMBC correlation to C-3 (δ_C_ 173.6), supporting the thiazole structure. The HMBC correlation of H-4 (δ_H_ 5.19) to C-3 extended the structure by a leucine unit, based on the COSY correlations of H-4 to 4-NH (δ_H_ 8.56) and to H-5 (δ_H_ 1.77) and of H-5 to H-6 (δ_H_ 1.65). H-6 showed COSY correlations to H-7 (δ_H_ 0.91) and H-8 (δ_H_ 0.87).

The adjacent *N*-Me alanine unit was connected based on the HMBC correlation of H-11 (δ_H_ 1.28) and 4-NH to C-9 (δ_C_ 170.6). Similarly, as observed and reported for **1**, broadening of the proton signals occurred, and the unit also did not show correlations in the HSQC spectrum [13].

The polyketide unit (C13–C29) was established as three spin systems with three trisubstituted double bonds and one disubstituted double bond that could be connected due to HMBC correlations. The first unit (C22–C25) was established from the COSY correlations of H-25 (δ_H_ 0.93) to H-24 (δ_H_ 2.07) and of H-24 to H-23 (δ_H_ 5.48), which belonged to a trisubstituted double bond. The HMBC from H-23 to C-29 (δ_C_ 12.1) and of H-29 (δ_H_ 1.63) to the non-protonated C-22 (δ_C_ 131.8) established that C-22 was methylated.

The COSY correlations of H-21 (δ_H_ 6.11) to H-20 (δ_H_ 5.26), of H-20 to H-19 (δ_H_ 3.91), of H-19 to H-18 (δ_H_ 2.43) and finally of H-18 to H-28 (δ_H_ 1.10) established a linear 5-carbon chain (methylated C18–C21), which was connected to the methylated C22–C25 unit as shown by HMBC correlation of H-29 to C-21 (δ_C_ 138.0). *E*-configuration of the C20–C21 double bond was confirmed by proton–proton coupling constant (*J* = 15.7 Hz).

The remaining α,β,γ,δ-unsaturated carbonyl system of the polyketide unit, consisting of two trisubstituted double bonds, was connected based on the HMBC correlations of H-18 and H-27 (δ_H_ 1.64) to the quaternary C-17 (δ_C_ 142.2). H-27 showed further HMBC correlations to C-16 (δ_C_ 121.0), and H-16 (δ_H_ 6.10) showed a COSY correlation to H-15 (δ_H_ 6.31), which in turn has an HMBC correlation to C-26 (δ_C_ 14.6). Finally, H-26 (δ_H_ 1.82) showed an HMBC correlation to C-14 (δ_C_ 130.4) and to C-13 (δ_C_ 173.1), which positioned the polyketide unit next to the *N*-Me alanine unit based on the HMBC correlation of H-12 (δ_H_ 2.78) to C-13. The configuration of the conjugated diene was established by NOESY (Figure 2).

The remaining unit was determined as a 3-*O*-methyl-methylpentose based on the COSY correlations of H-30 (δ_H_ 4.55) to H-31 (δ_H_ 3.75) and of that to H-32 (δ_H_ 3.12), which in turn had a COSY correlation to H-33 (δ_H_ 3.28), and that to H-34 (δ_H_ 3.48), which finally had a COSY correlation to H-35 (δ_H_ 1.13). The HMBC correlation of H-37 (δ_H_ 3.31) to C-32 (δ_C_ 80.7) guided the positioning of the *O*-Me, while the HMBC correlation of H-30 to C-19 (δ_C_ 78.0) completed the planar structure.

**Figure 4:**
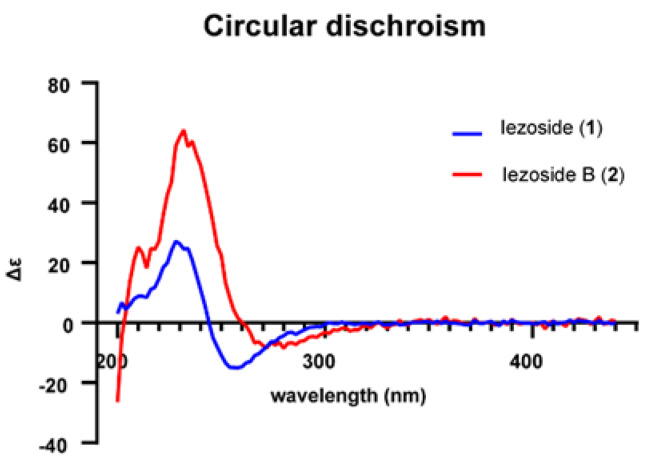
Circular dichroism data for iezoside (**1**) and iezoside B (**2**).

The configuration of C-20/C-21 and C-15/C-16 for both **1** and **2** was determined as trans and *s*-trans, respectively, based on the *J* coupling constants (15.7 Hz and 11.2 Hz, respectively). The absolute configuration of C-18/C-19 was determined by comparison of its circular dichroism (CD) spectra to the literature, as shown in Figure 4 [13]. Compound **2** shows a similar Cotton effect as **1**. Iezoside (**1**) shows a negative Cotton effect at 258 nm (Δε −3.6) and a positive at 234 nm (Δε +5.9), while iezoside B (**2**) shows a negative Cotton effect at 272 nm (Δε −1.2) and a positive at 232 nm (Δε +8.9). According to the published data for **1**, the C18/C19 configuration would be either 18*R*,19*R* or 18*S*,19*R* [13]. The NMR data for **1** was initially collected in DMSO-*d*_6_ and later compared and matched to the literature values for iezoside (**1**) in acetone-*d*_6_ as the planar structures and relative configuration of the compounds were identical (Figure S8, Table S1) [13]. By comparison of the ^1^H NMR spectra, the isolated compound matched the natural iezoside and not the 18*S*,19*R*-unnatural analogue; therefore, the absolute configuration was determined as 18*R*,19*R* [13]. The key differences between the two diastereomers were the H-27 and H-29 shifts as well as the upfield shift of the H-28 from δ_H_ 1.12 to δ_H_ 0.96. Coupling constants and NOESY data supported the relative configuration of the sugar moiety as rhamnose (Figure 2 and Figure 3), indicating the presence of 2,3-*O*-dimethyl-α-l-rhamnose in **1** and 3-*O*-methyl-α-l-rhamnose in **2**. For **1**, the sugar moiety was determined as rhamnose using the coupling constant between H-30 and H-31 (*J* 1.8 Hz) as well as the NOE correlations between H-31/H-34 and H-32/H-34. For **2**, the relative configuration was established using the coupling constant of H-34 to H-33 (*J* 11.8 Hz), and the NOE correlations between H-31/H-32 and H-31/H-30. H-30 is a broad singlet, indicating a small coupling constant to H-31 and further supporting the relative configuration. Amino acid analysis following ozonolysis and acid hydrolysis revealed L-Leu and *N*-Me-Ala as in published **1** [13]. Finally, the optical rotation ([α]^20^_D_ = +18.2 (c 0.05, CHCl_3_)) was compared to the reported data for **1** ([α]^25^_D_ = +56 (c 0.38, CHCl_3_)), [13] suggesting the same absolute configuration. The data for **2** ([α]^20^_D_ = +18.7 (c 0.013, CHCl_3_)) matched closely and in sign with those of **1**, suggesting **2** has the same absolute configuration as **1** [13].

### 2.2. Cytotoxicity and Effect on Intracellular Calcium

**Figure 5:**
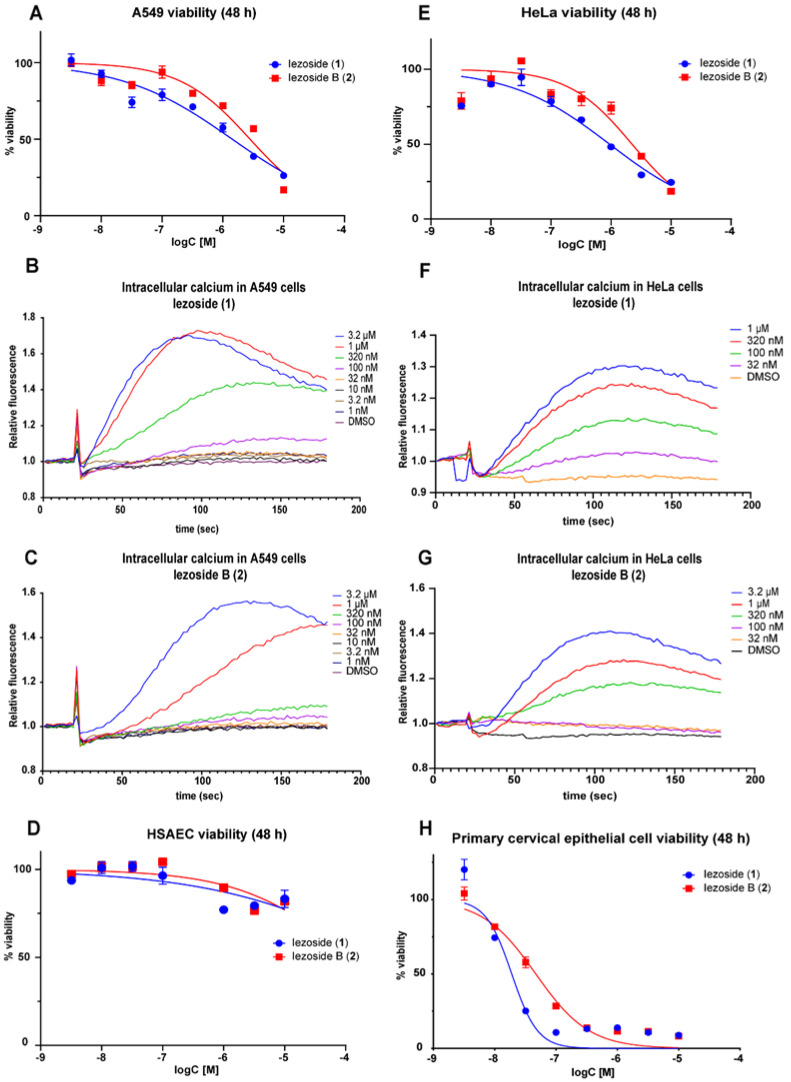
Biological activities of **1** and **2**. (**A**,**E**) Antiproliferative effect against A549 cells and against HeLa cells using the MTT assay after 48 h treatment. (**B**,**F**) Effect of **1** on intracellular calcium in A549 cells and HeLa cells. (**C**,**G**) Effect of **2** on intracellular calcium in A549 and HeLa cells. (**D**,**H**) Antiproliferative effect against HSAEC and against primary cervical epithelial cells using the MTT assay after 48 h treatment. All experiments were performed in *n* = 3.

Since compounds **1** and **2** were isolated from the same fraction as the cytotoxic lagunamide D (**3**)**,** they were originally tested for their cytotoxicity against A549 cells as described for **3** [23]. Iezoside (**1**) and B (**2**) were shown to possess micromolar activity against A549 cells. Iezoside (**1**) was shown to be two-fold more active than **2** with IC_50_ values of 1.5 and 3.0 μΜ, respectively (Figure 5A). Both compounds were shown to be non-cytotoxic against primary human small airway epithelial cells (HSAEC), showing over 70% viability at the highest tested concentration (10 μM) compared to the ~20% shown in the A549 line (Figure 5D), suggesting a potential preference for lung cancer cells over normal small airway epithelial cells. Thapsigargin showed a similar reduced cytotoxicity in HSAEC as shown in Figure S20.

The antiproliferative activity of **1** was originally observed in HeLa cells, concomitant with a change in morphology, an increase in cells in G1 phase and a decrease in cells in S phase, indicating a cell cycle delay [13]. Furthermore, **1** was tested in 39 human cancer cell lines by the Japanese Foundation for Cancer Research 39 (JFCR39), and the fingerprint showed similarities to that of thapsigargin and A23187. Iezoside (**1**) shows a similar phenotype in HeLa cells as thapsigargin and cyclopiazonic acid, both of which are SERCA inhibitors [14,16,17]. To further investigate that SERCA was the presumptive functional target of **1**, the compound was evaluated for its inhibitory effect of SERCA1a via a coupled enzyme assay and downstream effect on the expression of cell cycle regulation and apoptosis proteins [13].

With the prior knowledge of **1** being a SERCA inhibitor, both compounds were tested for their effect on cytosolic calcium oscillations in A549 cells, alongside thapsigargin. An increase in cytosolic calcium was observed near the IC_50_ for **1** and **2** (0.32 and 1 μΜ) with an approximate ΕC_50_ of 0.3 μM and 0.6 μM, respectively (Figure 5B,C, following the same trend as the antiproliferative activity for both compounds (Table 2).

**Figure 6:**
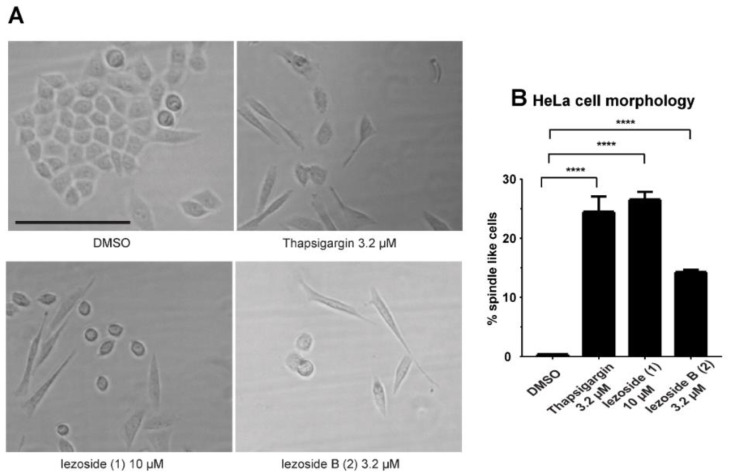
Effects on cell morphology. (**A**) Effect of **1**, **2** and thapsigargin on HeLa cell morphology after 24 h of treatment. Vehicle treatment (0.4 ± 0.0% of the cells show spindle like morphology), 3.2 μM of thapsigargin (24.5 ± 2.1% of the cells show spindle like morphology), 10 μΜ of **1** (26.5 ± 1.0% of the cells show spindle like morphology), 3.2 μΜ of **2** (14.3 ± 0.4% of the cells show spindle like morphology)**.** Scale bar 100 μm. (**B**) Quantification of morphological changes by visual scoring. Statistical analysis was performed using one-way ANOVA (**** *p* < 0.0001). All experiments were performed in triplicate (*n* = 3). For each individual experiment, three images/well were collected and counted with a total of 150–200 cells/image for **1**, **2** and thapsigargin, and 300–350 cells/image for the vehicle control.

The compounds were also tested for their cytotoxicity in HeLa cells, similarly as described in the literature [13]. The compounds showed an IC_50_ of 1.0 and 2.4 μΜ, respectively, at 48 h (Figure 5E), with compound **1** being 2.3-fold more potent than **2**. Our IC_50_ values are somewhat higher than reported (6.8 nM), although the precise experimental condition differed, including incubation time (72 h) and, possibly, genetic variations in the cell line [13]. Both compounds were shown to reduce cell viability of primary cervical epithelial cells with an IC_50_ of 0.02 and 0.05 μΜ, respectively (Figure 5H), while thapsigargin was two-fold more potent against primary cervical epithelial cells compared to HeLa cells (0.06 ± 0.04 μΜ in HeLa cells and 0.03 ± 0.01 μΜ in the primary cells). This data suggests substantial cell type-dependent differences and that the preference for cancer cells over normal cells cannot be generalized. The spindle-like morphology of HeLa cells that was previously observed for **1** was also noticeable in our experiments (Figure 6A), suggesting that **2** has a similar mechanism of action [13]. At the tested concentration, 26.5 ± 1.0% of the cells treated with **1** and 14.3 ± 0.4% of the cells treated with **2** showed the spindle-like morphology after 24 h of exposure (Figure 6B). In the calcium assay using HeLa cells, a similar fold difference was detected with activity at an approximate ΕC_50_ of 0.1 μM and 0.5 μM for **1** and **2**, respectively (Figure 5F,G, Table 2).

The effective concentration observed for both in the intracellular calcium assay is in the same order of magnitude as the viability assay and shows the same fold difference between the two compounds. These data indicate that the *O*-methyl in position C-31 is not essential for the activity of the compound class (Figure 5B,C,E,F) (Table 2), but that the C-31 *O*-Me group has incremental contribution to the overall activity at the level of target engagement and calcium flux as well as antiproliferative activity. This structure-activity relationship (SAR) suggests that the activity is potentially tunable through modification in the sugar moiety.

## 3. Materials and Methods

### 3.1. General Experimental Procedures

The optical rotations were recorded on a Rudolph Research Analytical Autopol III automatic polarimeter. NMR data were collected on a Bruker Avance II 600 MHz, high resolution 5-mm cryoprobe spectrometer operating at 600 MHz for ^1^H and 150 MHz for ^13^C, using residual solvent signals (δ_H_ 2.50; δ_C_ 39.5 ppm DMSO-*d*_6_, δ_H_ 2.50 ppm (CD_3_)_2_CO) as internal standards. The edited HSQC and HMBC experiments were optimized for ^1^*J*_CH_ = 140 Hz ^n^*J*_CH_ = 8 Hz, unless indicated otherwise (3 Hz HMBC). HRMS data were obtained using a Bruker Daltonics, Impact II QTOF with electrospray ionization (ESI). Chiral analysis was performed using an Agilent 6230 ESI-ToF and an Applied Biosystems 3200 QTRAP triple quad/linear trap. The effect on intracellular calcium was measured using a Molecular Devices Flexstation instrument. Circular Dichroism data were collected on a Chirascan™ Circular Dichroism Spectrometer (Applied Photophysics, Surrey, UK), with the Pro-Data Chirascan and Pro-Data viewer software 4.7.0.

### 3.2. Biological Material

The cyanobacterial tufts (DRTO 85) were collected from the shallow reef at Loggerhead Key, FL, on 9 May 2015. The cyanobacterial assemblage was microscopically identified as a mixture of *Dichothrix* sp., *Lyngbya* sp. and *Rivularia* sp. [23]. The Smithsonian Marine Station, Fort Pierce, FL, USA, maintains a voucher.

### 3.3. Extraction and Isolation

The cyanobacterium was lyophilized and extracted using 1:1 EtOAc:MeOH followed by 1:1 EtOH:H_2_O to provide 9.22 g and 11.88 g of extract, respectively. The extract was partitioned between EtOAc and H_2_O, followed by partitioning the polar layer between n-BuOH and H_2_O. The EtOAc fraction was subjected to a silica column using hexanes and increasing amounts of EtOAc, followed by increasing amounts of MeOH in EtOAC to give thirteen fractions. The SiO_2_ fraction eluting with 25% MeOH in EtOAc was further purified using a snap C18 column with increasing amounts of MeOH in H_2_O. The fraction eluting at 100% MeOH was further purified using a H_2_O/MeOH gradient (80–90% for 30 min, followed by 100% over 15 min) by HPLC (Phenomenex Synergi 4 μ Hydro-RP 80 Å, 250 × 10 mm, 4 μm; flow rate, 2.0 mL/min; UV detection at 200 nm and 220 nm), from which the fractions at *t*_R_ 18 min and *t*_R_ 24 min were further purified. The first fraction was purified using a H_2_O/ACN gradient (60–70% for 30 min, followed by 100% over 10 min) by HPLC (Phenomenex Synergi 4μ Hydro-RP 80 Å column, 250 × 10 mm, 4 μm UV detection at 200 nm, 2 mL/min) and yielded iezoside B (**2**) (0.2 mg) at *t*_R_ 25.5 min. The second fraction was further purified using a H_2_O/ACN gradient (60–80% for 30 min, followed by 100% over 10 min) by HPLC (Phenomenex Synergi 4μ Hydro-RP 80 Å column, 250 × 10 mm, 4 μm UV detection at 200 nm, 2 mL/min) which yielded iezoside (**1**) (0.8 mg) at *t*_R_ 30.1 min.

Iezoside (**1**): colorless oil, [α]^20^_D_ = +18.2 (c 0.05, CHCl_3_), reported [α]^25^_D_ = +56 (c 0.38, CHCl_3_), [13] CD (MeOH) Δε_234_ +5.9, Δε_258_ −3.6, ^1^H NMR, ^13^C NMR, COSY, and HMBC data in DMSO-*d*_6_, see Table 1; ^1^H NMR data in acetone-*d*_6_, see Table S1; HRESIMS *m*/*z* ([M+H]^+^ 690.4104 (calcd. for C_37_H_60_N_3_O_7_S, 690.4152).

Iezoside B (**2**): colorless oil, [α]^20^_D_ = +18.7 (c 0.013, CHCl_3_), CD (MeOH) Δε_232_ +8.9, Δε_272_ −1.2, ^1^H NMR, ^13^C NMR, COSY, and HMBC data in DMSO-*d*_6_ see Table 1; HRESIMS *m*/*z* ([M+H]^+^ 676.3990 (calcd. for C_36_H_58_N_3_O_7_S, 676.3954).

*Ozonolysis and acid hydrolysis:* A portion (50 μg) of **1** and **2** were dissolved in 3 mL of DCM, and O_3_ was bubbled in at −78 °C for 20 min. The samples were dried under nitrogen and were subjected to oxidative workup (2:1 FA:H_2_O_2_, 70 °C, 30 min). The samples were dried under vacuum, subjected to acid hydrolysis (6 N HCl, 110 °C, 18 h) and then evaporated to dryness. The samples were reconstituted in 50 μL H_2_O and subjected to chiral analysis using three separate conditions. Condition 1: (Chirobiotic TAG (4.6 mm × 250 mm), Supelco; solvent, MeOH −10 mM NH4OAc (40:60); flow rate, 0.5 mL/min; detection by ESIMS in positive mode)]. The retention times (*t*_R_, min) were as follows: L-Leucine (8.7 min). Both *N*-Me-L-Alanine (9.9 min) and *N*-Me-D-Alanine (47.0 min) were observed at a 1:5 ratio, indicating the presence of *N*-Me-D-Alanine. The authentic standards eluted at L-Leucine (8.7 min), D-Leucine (15.21 min), *N*-Me-L-Alanine (9.9 min), *N*-Me-D-Alanine (47.0 min).

### 3.4. Cell Viability Assay

A549 (American Type Culture Collection, ATCC), HSAEC (ATCC), HeLa (ATCC) and primary cervical epithelial (ATCC) cells were cultured in Dulbecco’s modified Eagle’s medium (DMEM) supplemented with 10% fetal bovine serum at 37 °C humidified air and 5% CO_2_. Cells were seeded (7500/well (A549, HSAEC) and 2000/well (HeLa, primary cervical epithelial cells)) in 96-well plates and allowed to attach overnight before being treated with the compounds or the solvent control (0.5% DMSO). The cells were incubated for 48 h followed by addition of MTT dye, according to the manufacturer’s protocol (Promega). IC_50_ values were calculated using GraphPad prism software 9.2.0. 

### 3.5. HeLa Cell Morphology Study

HeLa (ATCC) cells were cultured in Dulbecco’s modified Eagle’s medium (DMEM) supplemented with 10% fetal bovine serum at 37 °C humidified air and 5% CO_2_. Cells were seeded 700/well in 384-well plates and allowed to attach overnight before being treated with the compounds or the solvent control (0.5% DMSO). The cells were incubated for 24 h followed by observation under the microscope to quantify the morphological changes.

### 3.6. Intracellular Calcium Assay

A549 and HeLa cells were seeded (10,000/well and 20,000/well, respectively) overnight in black plates. The cells were allowed to attach overnight before the compounds or the solvent control (0.5% DMSO) was added. The FLIPR calcium 6 assay kit was used following the manufacturer’s protocol (FLIPR Ca 6, Molecular Devices, San Jose, CA, USA). For the calculation of the approximate EC_50_ values, the vehicle control was used as the minimum response, and the maximum detected response before saturating the detector was used as the maxima.

## 4. Conclusions

In conclusion, we report the isolation of iezoside (**1**) and iezoside B (**2**), the second member of the peptide-polyketide hybrid glycoside, from a collection of a marine cyanobacterial assemblage from Florida. Iezoside (**1**) was previously isolated from Okinawa, Japan, and now isolated from the Dry Tortugas, Florida, indicating the broad distribution of the compound. Lagunamide D (**3**), also isolated from the same extract, was proven to be a mitochondrial cytotoxin, which further supports the richness of cyanobacterial extracts [24]. Compound **2** shows similar but slightly weaker activity than **1** in the assays performed, indicating that the methylation on the sugar moiety only minorly impacts the biological effect. The SAR provides evidence that structural changes in the sugar unit may be tolerated and that the activity of this compound class is tunable. This finding has implications for future analogue synthesis and potential target interaction studies.

## Figures and Tables

**Table 1:** NMR data for iezoside and iezoside B (**2**) in DMSO-*d*_6_.

Iezoside (1)						Iezoside B (2)				
Position	δ_H_, mult., *J* (Hz) ^a^	δ_C_ ^b^	HMBC	COSY	NOESY	δ_H_, mult., *J* (Hz) ^a^	δ_C_ ^b^	HMBC	COSY	NOESY
1	7.70, d (3.3)	142.1	2, 3	2		7.70, d (3.3)	142.2	2, 3	2	
2	7.58, d (3.3)	119.1	1	1		7.58, d (3.3)	119.4		1	
3		173.6					173.6			
4	5.19, m	49.2	3, 5	5, 8-NH		5.19, m	49.2	3, 5	5, 8-NH	
5	1.77, m	43.2	4	4, 6		1.77, m	43.2	3, 4, 6, 7, 8	4, 6	
6	1.65, m	24.4		7, 8		1.65, m	24.3		6, 7, 8	
7	0.90, d (6.7)	23.0	5, 6, 8	6		0.91, d (7.0)	22.9	5, 6, 8	6	
8	0.87, d (6.5)	21.4	5, 6, 7	6		0.87, d (6.5)	21.3	5, 6, 7	6	
NH	8.58, d (8.4)		9	4		8.56, d (8.5)		9	4	
9		170.5					170.6			
10	4.83, br	^c^		11		4.80, br	^c^			
11	1.29, br d (7.2)	^c^	9	10		1.28, br d (7.1)	^c^	9		12
12	2.78, s	^c^	13			2.78, s	^c^	13		11
13		173.0					173.1			
14		130.2					130.4			
15	6.32, d (11.2)	121.0	13, 17, 26	16	27	6.31, d (11.2)	121.0	13, 26	16	27
16	6.09, d (11.2)	121.1	14, 18, 27	15	18, 26, 28	6.10, d (11.2)	121.0	18, 27	15	18, 26
17		142.6					142.2			
18	2.43, p (6.7)	47.7	16, 17, 19, 20	19, 28		2.43, p (6.7)	47.7	16, 17, 19, 28	19, 28	16
19	3.95, t (8.0)	78.2	18, 21, 28, 30	18, 20	27	3.91, t (8.0)	78.0	18, 21, 28, 30	18, 20	21, 27, 28, 30
20	5.29, dd (15.7, 8.4)	124.4	22	19, 21	29	5.26, dd (15.7, 8.4)	124.6	22	19, 21	29
21	6.14, d (15.7)	138.1	19, 22, 23, 29	20		6.11, d (15.7)	138.0	19, 22, 23, 29	20	19
22		131.8					131.8			
23	5.49, t (7.2)	134.7	21, 24, 29	24	24	5.48, t (7.2)	134.9	21, 24, 25, 29	24	24
24	2.08, m	21.0	22, 23, 25	23, 25		2.07, m	20.9	22, 23, 25	23, 25	23, 29
25	0.93, t (7.5)	14.0	23, 24	24		0.93, t (7.5)	13.8	23, 24	24	
26	1.83, s	14.3	13, 14			1.82, s	14.6	13, 14		16
27	1.65, s	14.8	16, 17, 18			1.64, s	14.3	16, 17, 18		15, 19
28	1.10, d (7.0)	15.4	17, 18, 19	18		1.10, d (7.0)	15.5	17, 18, 19	18	
29	1.64, s	12.1	21, 22			1.63, s	12.1	21, 22, 23	20	24
30	4.69, d (1.8)	93.0	19, 32, 34	31	31	4.55, br s	96.2	19, 31, 32, 34	31	19, 31
31	3.47, m	76.7	32, 33, 36	30	30	3.75, m	66.6		30, 32	30, 32
31-OH	-					4.71, d (4.5)		30, 31, 32		37
32	3.21, m	81.0	31		34	3.12, dd (9.3, 3.2)	80.7	33, 36	31, 33	31
33	3.21, m	70.9				3.28, m	70.6	34, 35	32, 34	
33-OH	4.97, br					4.87, d (5.5)		33, 34, 35		
34	3.47, m	69.0	32, 33	35	32, 36	3.48, dq (11.8, 6.0)	68.9		33, 35	35
35	1.12, d (6.2)	18.0	33, 34	34		1.13, d (6.0)	17.9	33, 34	34	34
36	3.27, m	58.3	31		34	-				
37	3.32, m	56.8	32			3.31, m	56.2	32		

^a^ Recorded at 600 MHz; ^b^ Recorded at 150 MHz; ^c^ These signals were not detected.

**Table 2:** Cell viability IC_50_ values and approximate EC_50_ values for intracellular calcium flux for iezoside (**1**), iezoside B (**2**) in μΜ.

	A549		HeLa		HSAEC	Primary Cervical Epithelial Cells
	IC_50_	EC_50_	IC_50_	EC_50_	IC_50_	IC_50_
Iezoside (**1**)	1.5 ± 0.5	~0.3	1.0 ± 0.4	~0.1	>10	0.02 ± 0.01
Iezoside B (**2**)	3.0 ± 0.9	~0.6	2.4 ± 0.9	~0.5	>10	0.05 ± 0.01

## Data Availability

Data is contained within the article or supplementary materials. Raw data will be made available upon request.

## Supplementary Materials

The following supporting information can be downloaded at: https://www.mdpi.com/article/10.3390/md21070378/s1, Figures S1–S15: 1D/2D NMR data of iezoside and iezoside B in DMSO-*d*_6_ and acetone-*d*_6_; Figures S16–S21: Bioassay data for thapsigargin, Table S1: Comparison of experimental and literature ^1^H NMR data of iezoside in acetone-*d*_6_.

## References

[1] Carafoli E. (1987). Intracellular Calcium Homeostasis. Annu. Rev. Biochem..

[2] Carafoli E. (2002). Calcium signaling: A tale for all seasons. Proc. Natl. Acad. Sci. USA.

[3] Blaustein M.P., Lederer W.J. (1999). Sodium/calcium exchange: Its physiological implications. Physiol. Rev..

[4] Denmeade S.R., Isaacs J.T. (2005). The SERCA pump as a therapeutic target: Making a “smart bomb” for prostate cancer. Cancer Biol. Ther..

[5] Chemaly E.R., Troncone L., Lebeche D. (2018). SERCA control of cell death and survival. Cell Calcium.

[6] Carroll A.R., Copp B.R., Davis R.A., Keyzers R.A., Prinsep M.R. (2021). Marine natural products. Nat. Prod. Rep..

[7] Salvador-Reyes L.A., Luesch H. (2015). Biological targets and mechanisms of action of natural products from marine cyanobacteria. Nat. Prod. Rep..

[8] Mi Y., Zhang J., He S., Yan X. (2017). New peptides isolated from marine cyanobacteria, an overview over the past decade. Mar. Drugs.

[9] Teruya T., Sasaki H., Kitamura K., Nakayama T., Suenaga K. (2009). Biselyngbyaside, a macrolide glycoside from the marine Cyanobacterium *Lyngbya* sp. Org. Lett..

[10] Morita M., Ohno O., Teruya T., Yamori T., Inuzuka T., Suenaga K. (2012). Isolation and structures of biselyngbyasides B, C, and D from the marine cyanobacterium *Lyngbya* sp., and the biological activities of biselyngbyasides. Tetrahedron.

[11] Iwasaki A., Ohno O., Sumimoto S., Suda S., Suenaga K. (2014). Kurahyne, an acetylene-containing lipopeptide from a marine cyanobacterial assemblage of *Lyngbya* sp. RSC Adv..

[12] Iwasaki A., Ohno O., Katsuyama S., Morita M., Sasazawa Y., Dan S., Simizu S., Yamori T., Suenaga K. (2015). Identification of a molecular target of kurahyne, an apoptosis-inducing lipopeptide from marine cyanobacterial assemblages. Bioorganic Med. Chem. Lett..

[13] Kurisawa N., Iwasaki A., Teranuma K., Dan S., Toyoshima C., Hashimoto M., Suenaga K. (2022). Structural Determination, Total Synthesis, and Biological Activity of Iezoside, a Highly Potent Ca^2+^-ATPase Inhibitor from the Marine Cyanobacterium *Leptochromothrix valpauliae*. J. Am. Chem. Soc..

[14] Lytton J., Westlin M., Hanley M.R. (1991). Thapsigargin inhibits the sarcoplasmic or endoplasmic reticulum Ca-ATPase family of calcium pumps. J. Biol. Chem..

[15] Sehgal P., Szalai P., Olesen C., Praetorius H.A., Nissen P., Christensen S.B., Engedal N., Møller J.V. (2017). Inhibition of the sarco/endoplasmic reticulum (ER) Ca2-ATPase by thapsigargin analogs induces cell death via ER Ca2 depletion and the unfolded protein response. J. Biol. Chem..

[16] Seidler N.W., Jona I., Vegh M., Martonosi A. (1989). Cyclopiazonic acid is a specific inhibitor of the Ca^2+^-ATPase of sarcoplasmic reticulum. J. Biol. Chem..

[17] Uyama Y., Imaizumi Y., Watanabe M. (1993). Cyclopiazonic acid, an inhibitor of Ca^2+^-ATPase in sarcoplasmic reticulum, increases excitability in ileal smooth muscle. Br. J. Pharmacol..

[18] Doan N.T.Q., Paulsen E.S., Sehgal P., Møller J.V., Nissen P., Denmeade S.R., Isaacs J.T., Dionne C.A., Christensen S.B. (2015). Targeting thapsigargin towards tumors. Steroids.

[19] Jaskulska A., Janecka A.E., Gach-Janczak K. (2020). Thapsigargin—From Traditional Medicine to Anticancer Drug. Int. J. Mol. Sci..

[20] Isaacs J.T., Brennen W.N., Christensen S.B., Denmeade S.R. (2021). Mipsagargin: The beginning—Not the end—Of thapsigargin prodrug-based cancer therapeutics. Molecules.

[21] Andersen T., López C., Manczak T., Martinez K., Simonsen H. (2015). Thapsigargin—From *Thapsia* L. to Mipsagargin. Molecules.

[22] Morita M., Ogawa H., Ohno O., Yamori T., Suenaga K., Toyoshima C. (2015). Biselyngbyasides, cytotoxic marine macrolides, are novel and potent inhibitors of the Ca^2+^ pumps with a unique mode of binding. FEBS Lett..

[23] Luo D., Putra M.Y., Ye T., Paul V.J., Luesch H. (2019). Isolation, Structure Elucidation and Biological Evaluation of Lagunamide D: A New Cytotoxic Macrocyclic Depsipeptide from Marine Cyanobacteria. Mar. Drugs.

[24] Luo D., Ratnayake R., Atanasova K.R., Paul V.J., Luesch H. (2023). Targeted and functional genomics approaches to the mechanism of action of lagunamide D, a mitochondrial cytotoxin from marine cyanobacteria. Biochem. Pharmacol..

