# Supplementary material for: Isolation and Biological Activity of Iezoside and Iezoside B, SERCA Inhibitors from Floridian Marine Cyanobacteria
Source: Mar Drugs. 2023 Jun 27;21(7):378. doi: 10.3390/md21070378 (PMC10381893; doi:10.3390/md21070378)
Supplement: Supplementary file 1 [file marinedrugs-21-00378-s001.zip › marinedrugs-2452482-supplementary.pdf]

Supplementary Materials:

Isolation and Biological Activity of Iezoside and Iezoside B, SERCA Inhibitors from Floridian Marine Cyanobacteria

Sofia Kokkaliari [1], Danmeng Luo [1], Valerie J. Paul [2] and Hendrik Luesch [1,*]

[1] Department of Medicinal Chemistry and Center for Natural Products, Drug Discovery and Development (CNPD3), University of Florida, 1345 Center Drive, Gainesville, FL 32610, USA

[2] Smithsonian Marine Station at Ft. Pierce, 701 Seaway Drive, Fort Pierce, FL 34949, USA

* Correspondence: luesch@cop.ufl.edu; Tel.: +(352)-273-7738

Contents:

Figure S1. ^{1}H NMR of iezoside (**1**) in DMSO-d_6 (600 MHz) ... S2
Figure S2. ^{13}C NMR of iezoside (**1**) in DMSO-d_6 (150 MHz) ... S3
Figure S3. COSY NMR of iezoside (**1**) in DMSO-d_6 (600 MHz) ... S4
Figure S4. HSQC of iezoside (**1**) in DMSO-d_6 (600 MHz) ... S5
Figure S5. HMBC of iezoside (**1**) in DMSO-d_6 (600 MHz) ... S6
Figure S6. 3Hz HMBC of iezoside (**1**) in DMSO-d_6 (600 MHz) ... S7
Figure S7. NOESY of iezoside (**1**) in DMSO-d_6 (600 MHz) ... S8
Figure S8. ^{1}H NMR of iezoside (**1**) in acetone-d_6 (600 MHz) ... S9
Table S1. ^{1}H NMR comparison of iezoside (**1**) in acetone-d_6 (600 MHz) ... S10
Figure S9. ^{1}H NMR of iezoside B (**2**) in DMSO-d_6 (600 MHz) ... S11
Figure S10. ^{13}C NMR of iezoside B (**2**) in DMSO-d_6 (150 MHz) ... S12
Figure S11. COSY of iezoside B (**2**) in DMSO-d_6 (600 MHz) ... S13
Figure S12. HSQC of iezoside B (**2**) in DMSO-d_6 (600 MHz) ... S14
Figure S13. HMBC of iezoside B (**2**) in DMSO-d_6 (600 MHz) ... S15
Figure S14. TOCSY of iezoside B (**2**) in DMSO-d_6 (600 MHz) ... S16
Figure S15. NOESY of iezoside B (**2**) in DMSO-d_6 (600 MHz) ... S17
Figure S16. Dose-response curve (cell viability) for thapsigargin in A549 cells at 48 h ... S18
Figure S17. Effect of thapsigargin on cytosolic calcium in A549 cells ... S18
Figure S18. Dose-response curve (cell viability) for thapsigargin in HeLa cells at 48 h ... S18
Figure S19. Effect of thapsigargin in the cytosolic calcium in HeLa cells ... S19
Figure S20. Dose-response curve (cell viability) for thapsigargin in HSAEC cells at 48 h ... S19
Figure S21. Dose-response curve (cell viability) for thapsigargin in primary cervical epithelial cells ... S19
Reference ... S19

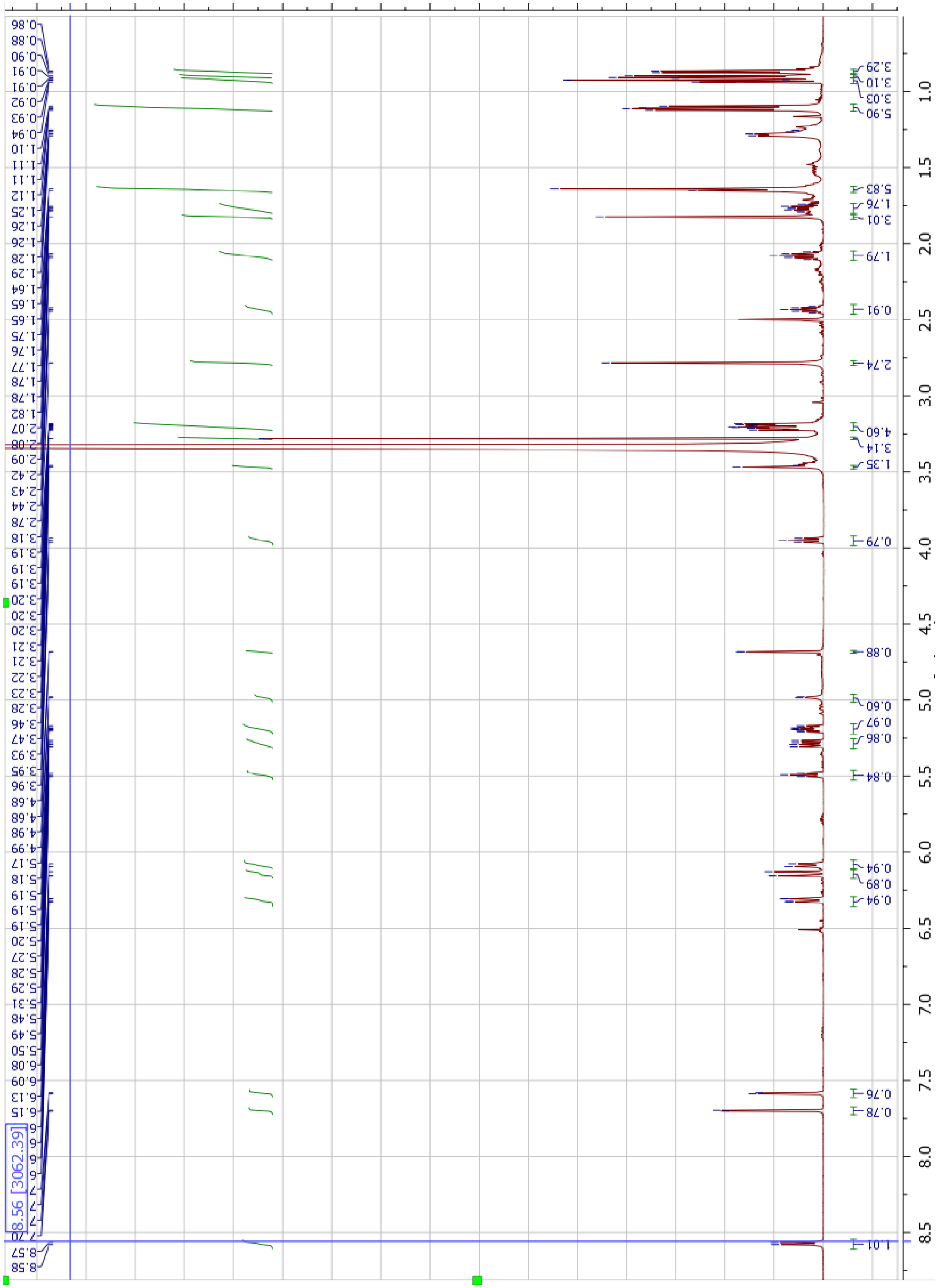

Figure S1. ^{1}H NMR of iezoside (**1**) in DMSO-d_6.

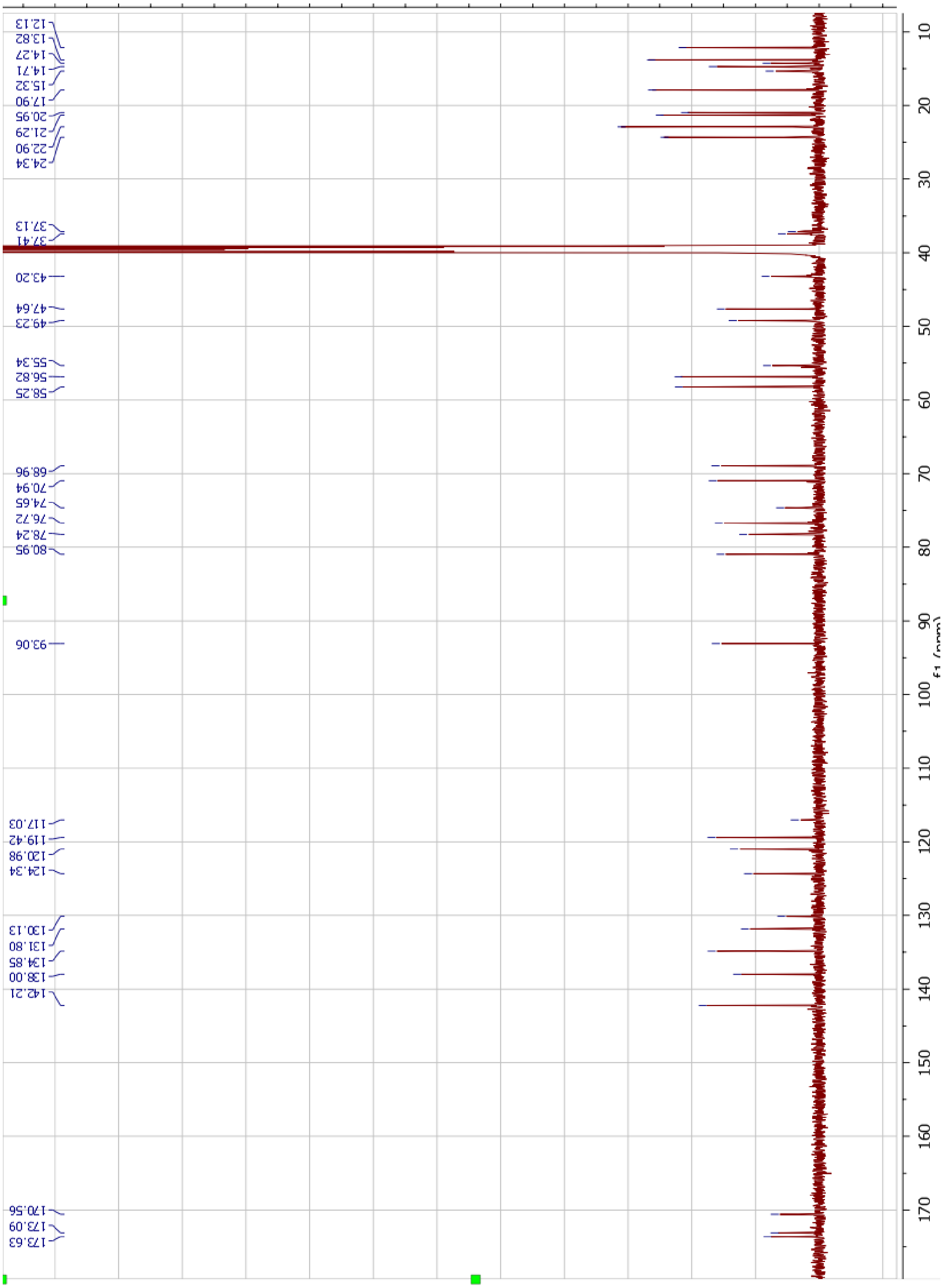

Figure S2. ^{13}C NMR of iezoside (**1**) in DMSO-d_6.

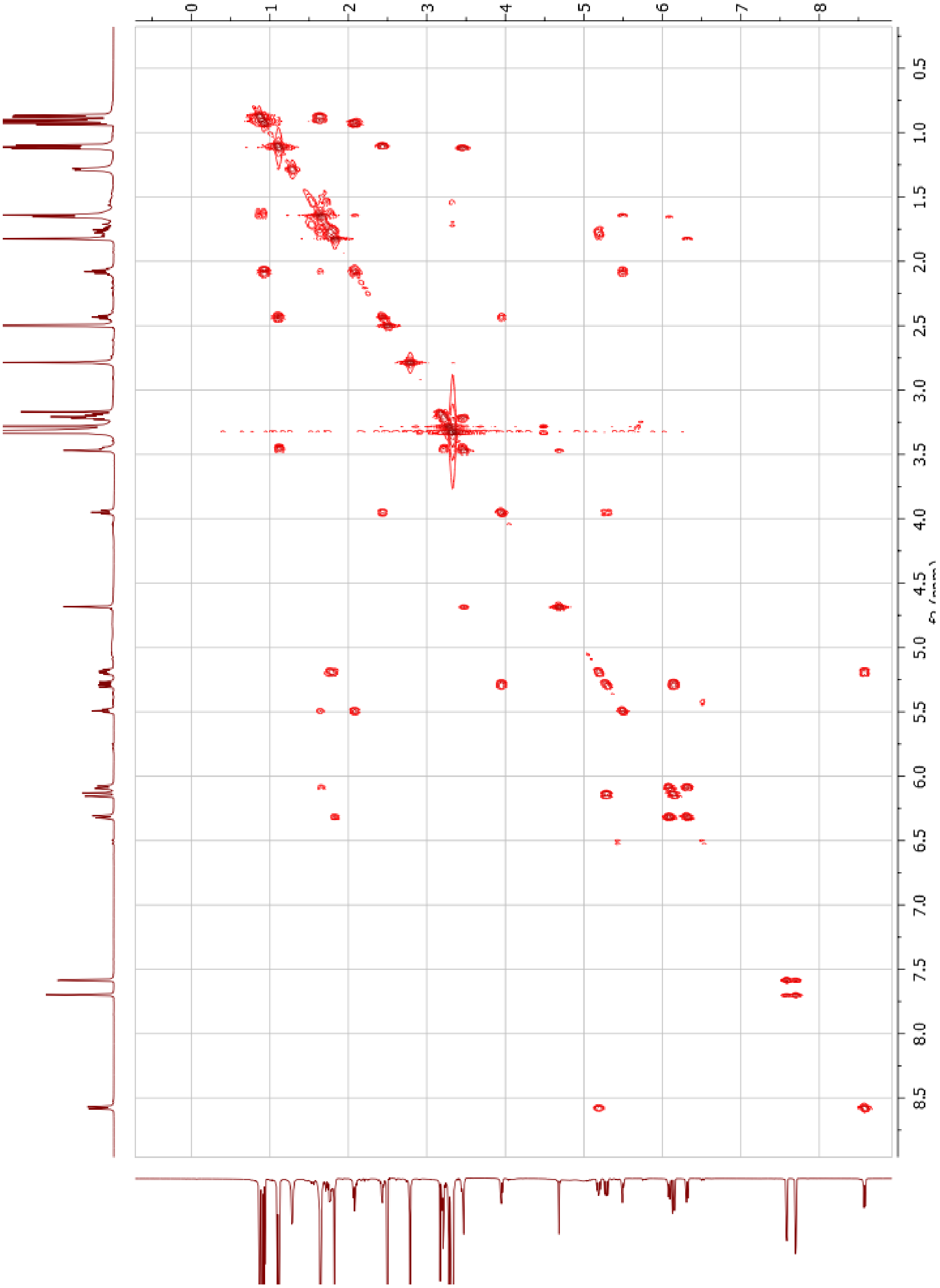

Figure S3. COSY NMR of iezoside (**1**) in DMSO-d_6.

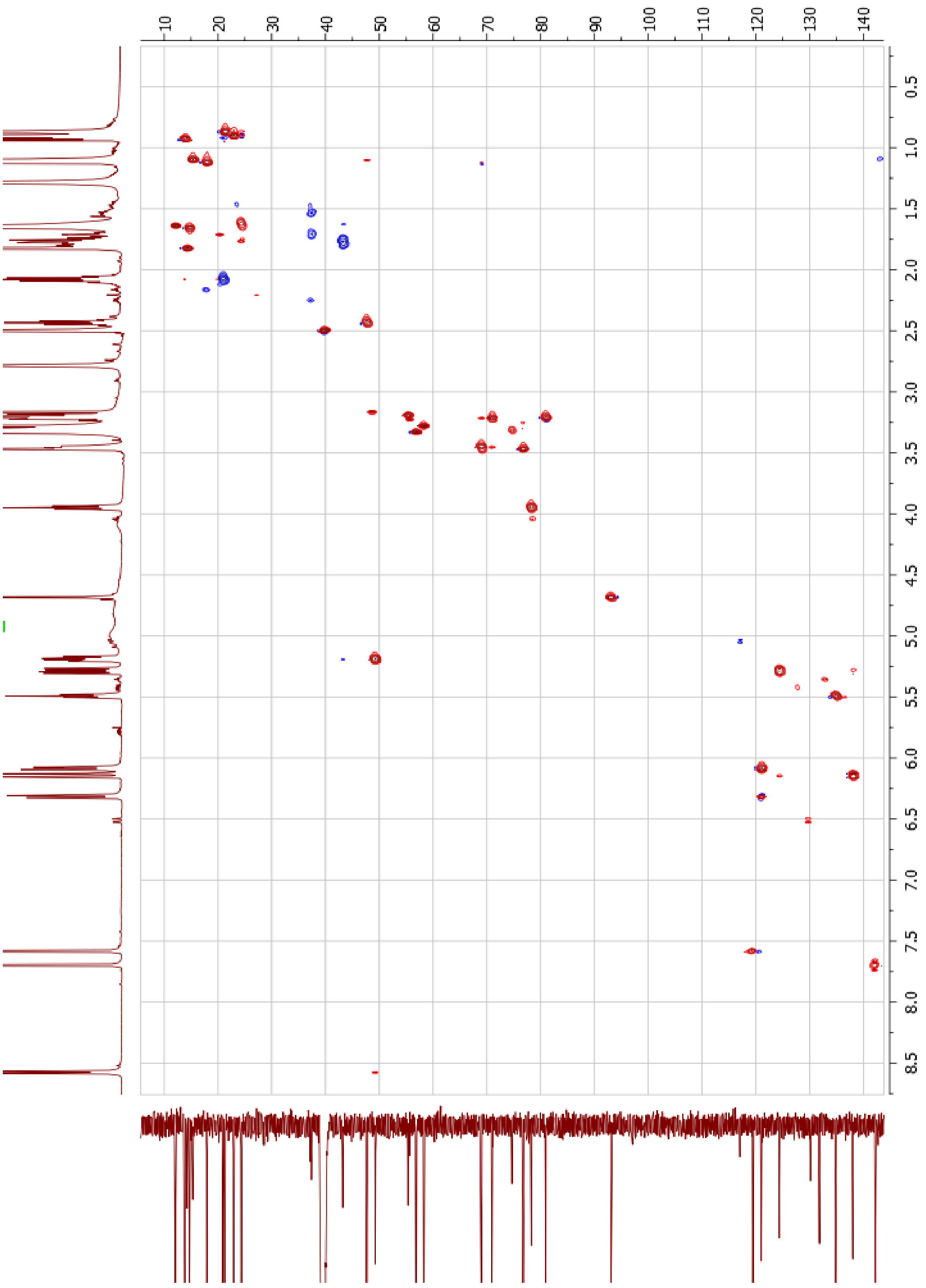

Figure S4. HSQC of iezoside (**1**) in DMSO-d_6.

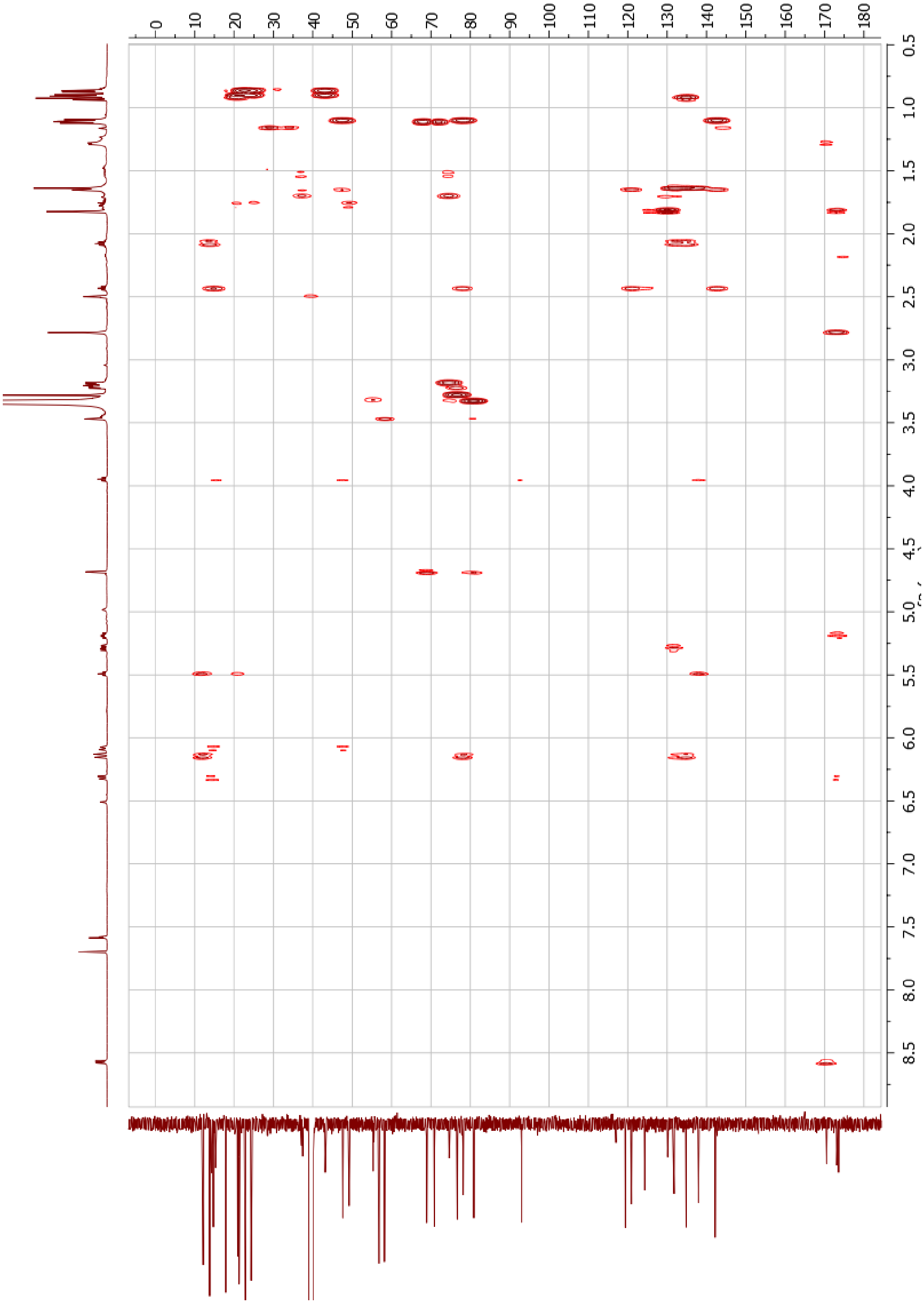

Figure S5. HMBC of iezoside (**1**) in DMSO-d_6.

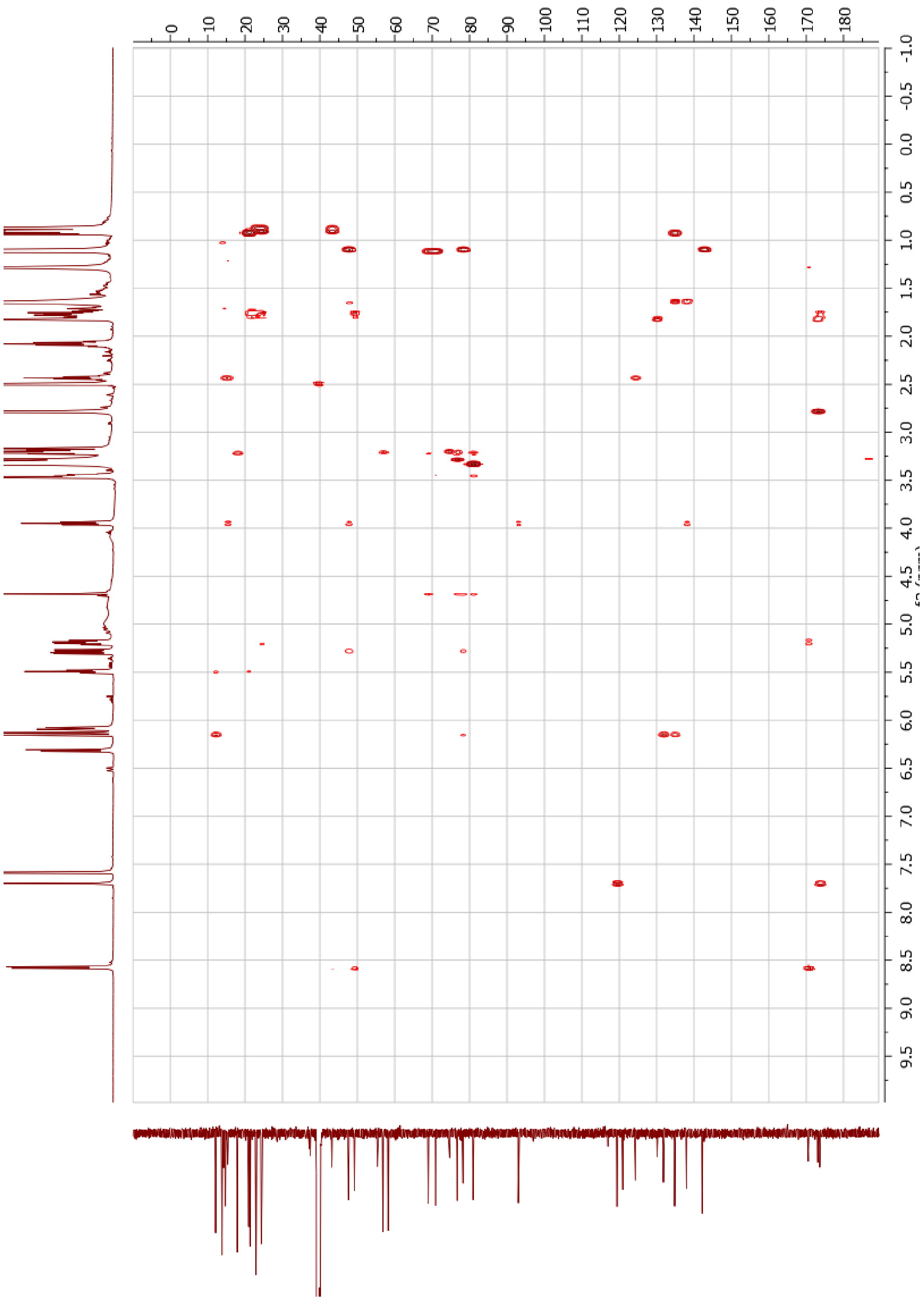

Figure S6. 3Hz HMBC of iezoside (**1**) in DMSO-d_6.

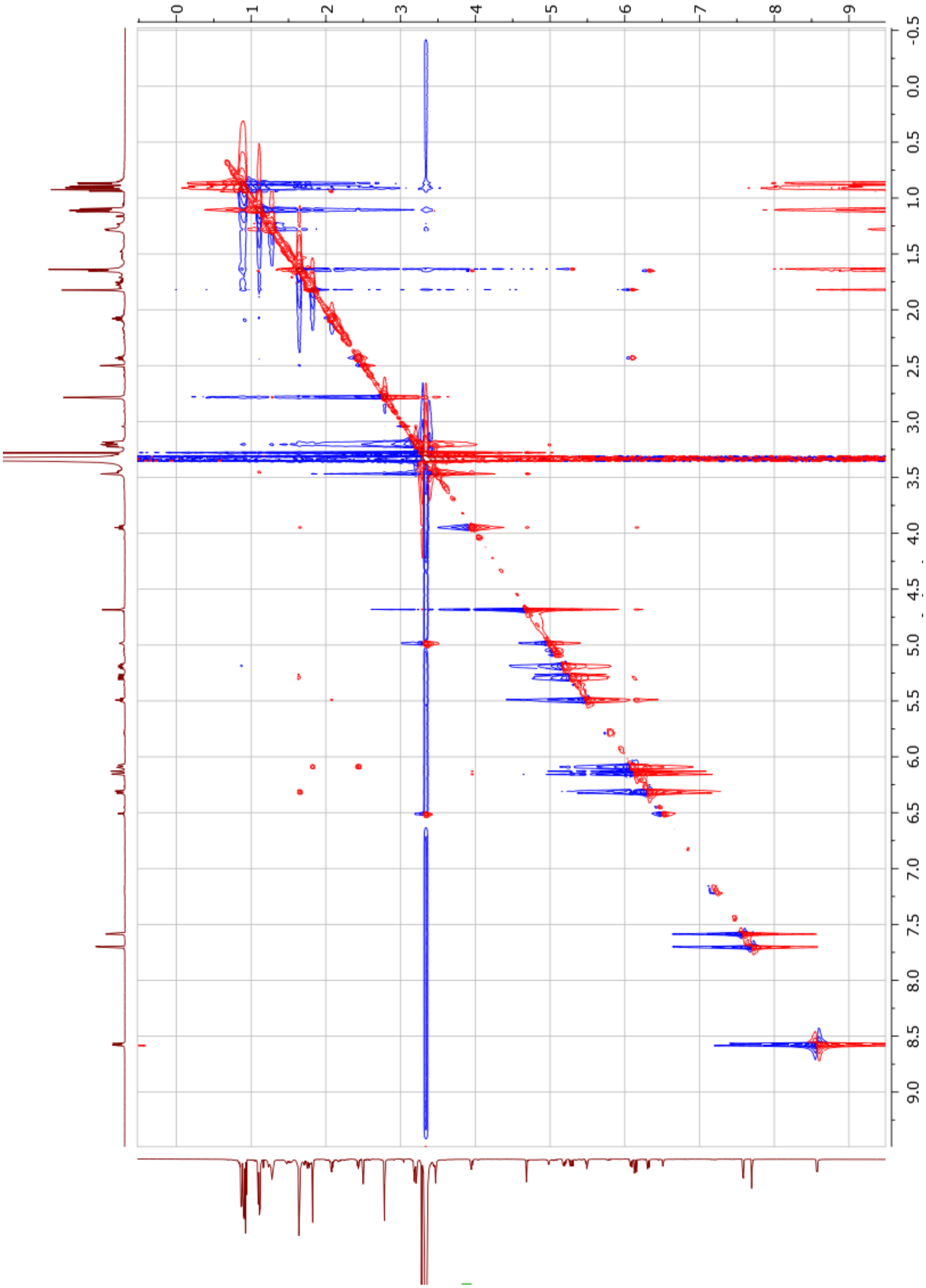

Figure S7. NOESY of iezoside (**1**) in DMSO-*d*$_6$.

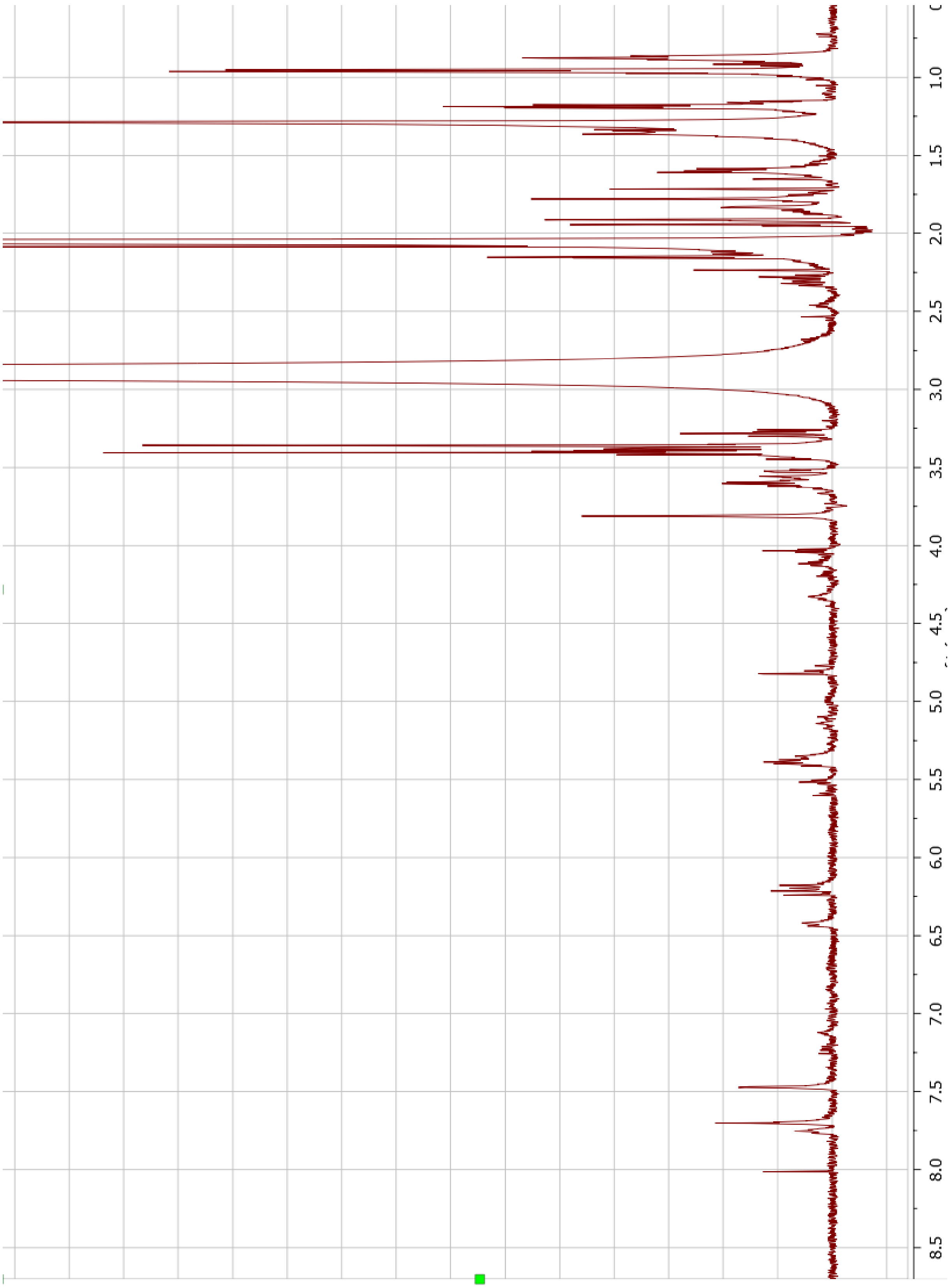

Figure S8. ^{1}H NMR of iezoside (**1**) in acetone-d_6.

Table S1. ^{1}H NMR comparison of Iezoside (**1**) in Acetone-d_6

	Reported[1]	Experimental
Position	δ_H, mult., J (Hz)[a]	δ_H, mult., J (Hz)[a]
1	7.70, d (3.2)	7.70, d (3.3)
2	7.48, d (3.2)	7.47, d (3.3)
3		
4	5.40, m	5.40, m
5	1.85, m	1.85, m
6	1.77, m	1.77, m
7	0.96, d (6.4)	0.96, d (6.4)
8	0.96, d (6.4)	0.96, d (6.4)
NH	7.74, d (8.5)	
9		
10	4.99, br	4.99, br
11	1.35, d (6.7)	1.35, d (6.1)
12	2.86, s	2.79, s
13		
14		
15	6.43, d (11.3)	6.43, d (11.6)
16	6.19, d (11.3)	6.19, d (11.8)
17		
18	2.46, dq (6.5, 6.9)	2.46, m
19	4.12 dd (6.5, 8.6)	4.12 dd (6.5, 8.7)
20	5.40, dd (8.6, 16.0)	5.40, m
21	6.23, d (16.0)	6.23, d (15.7)
22		
23	5.52, t (7.7)	5.52, t (7.6)
24	2.14, dq (7.7, 7.5)	2.14, m
25	0.96, t (7.5)	0.96, m
26	1.91, d (0.9)	1.91, d (1.0)
27	1.78, s	1.78, s
28	1.18, d (6.9)	1.18, d (6.8)
29	1.72, s	1.72, s
30	4.82, d (1.7)	4.82, d (1.8)
31	3.56, dd (1.7, 2.9)	3.60, m
32	3.37, dd (2.9, 8.8)	3.37, m
33	3.44, dd (8.8, 9.3)	3.45, m
34	3.60, qd (6.3, 9.3)	3.60, m
35	1.19, d (6.3)	1.19, d (6.2)
36	3.36, s	3.36, s
37	3.40, s	3.40, s

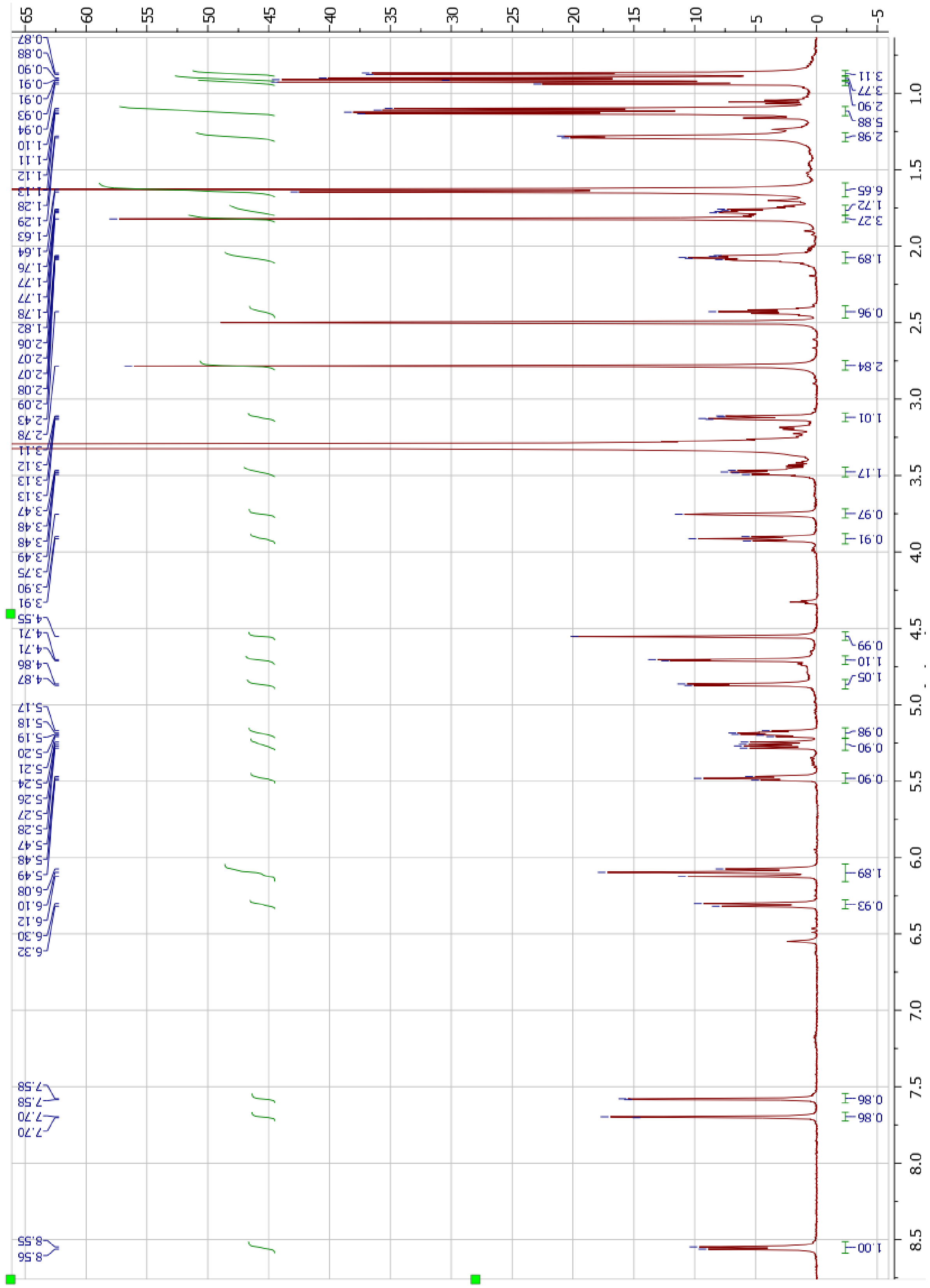

Figure S9. ^{1}H NMR of iezoside B (**2**) in DMSO-d_6.

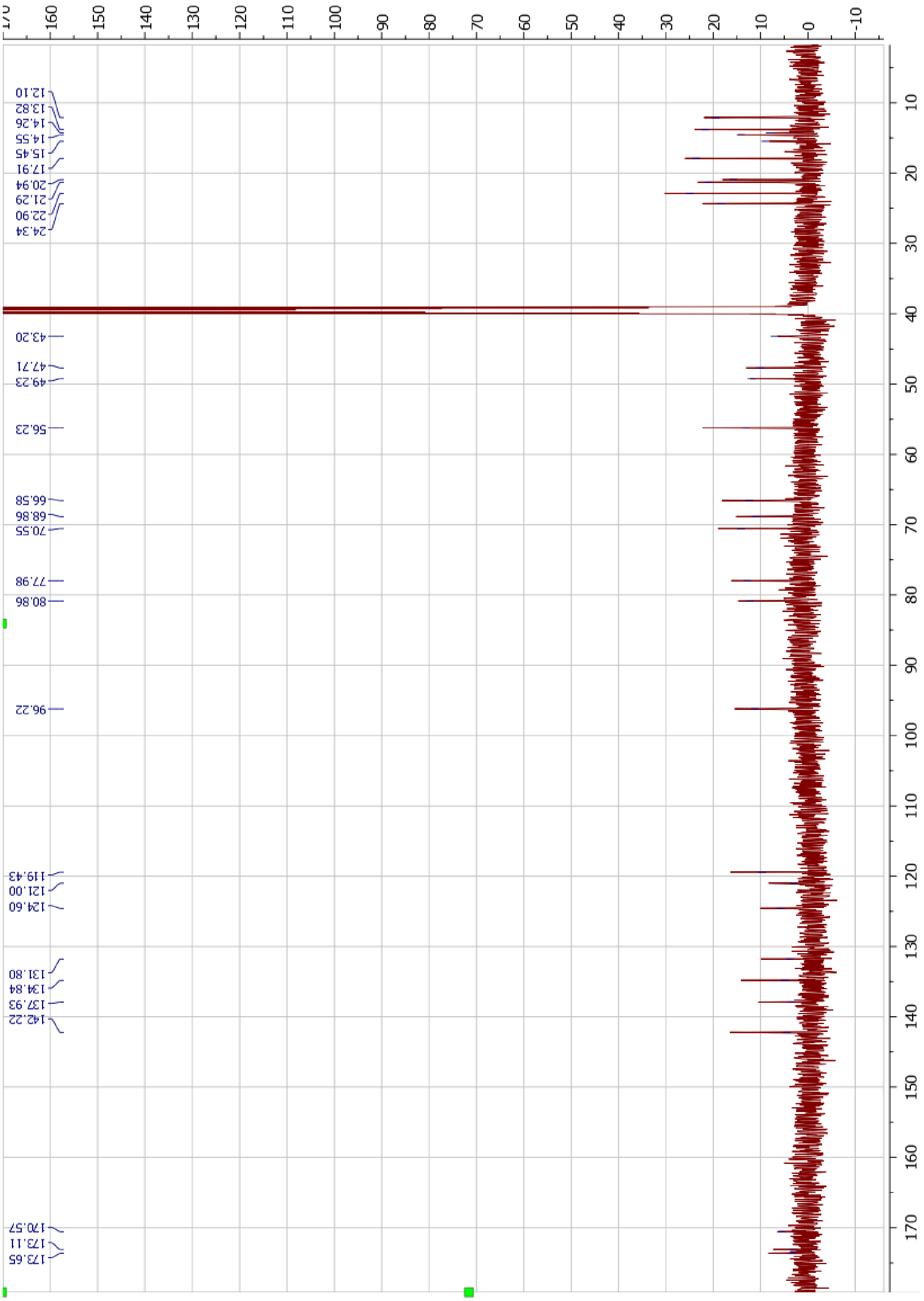

Figure S10. ^{13}C NMR of iezoside B (**2**) in DMSO-d_6.

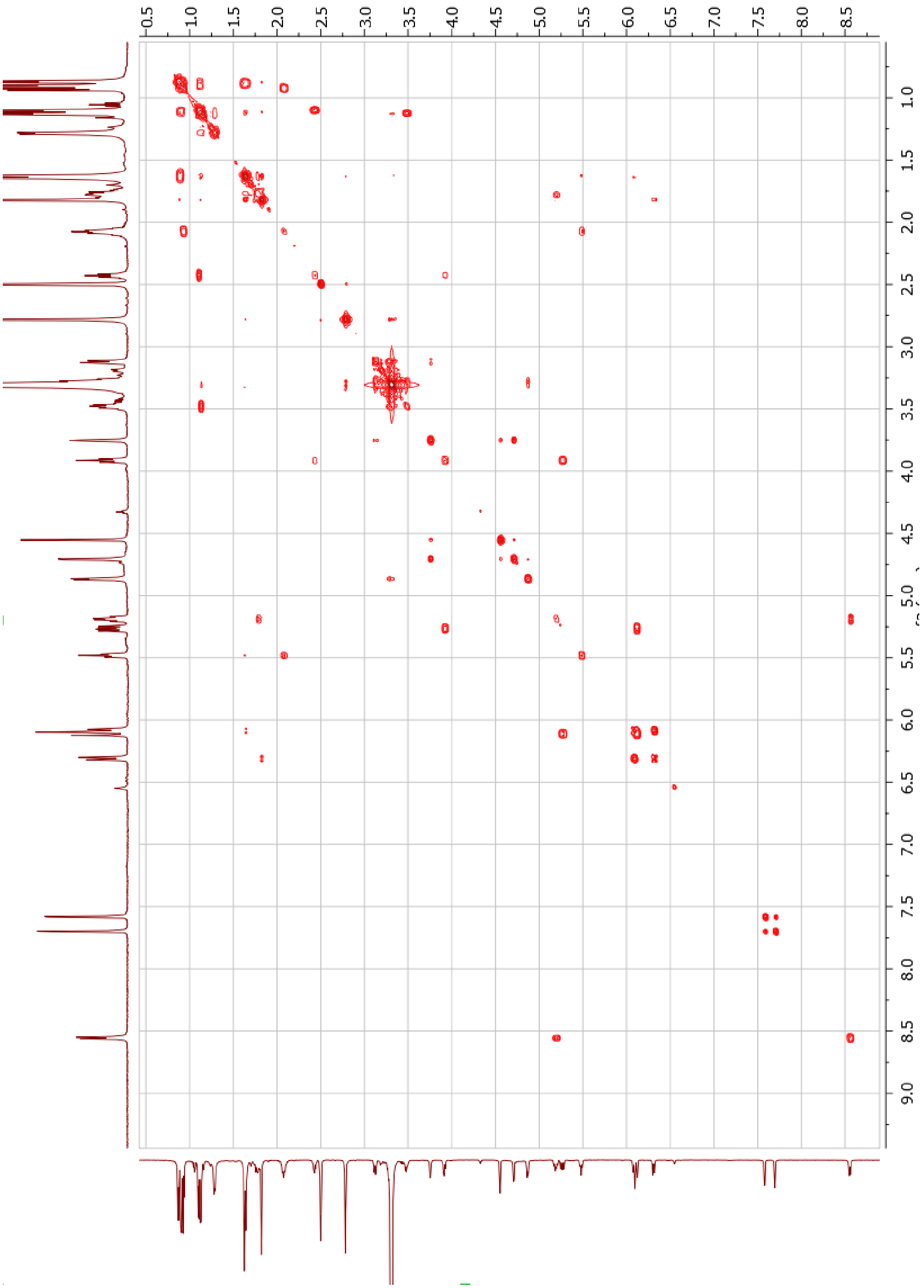

Figure S11. COSY of iezoside B (**2**) in DMSO-d_6.

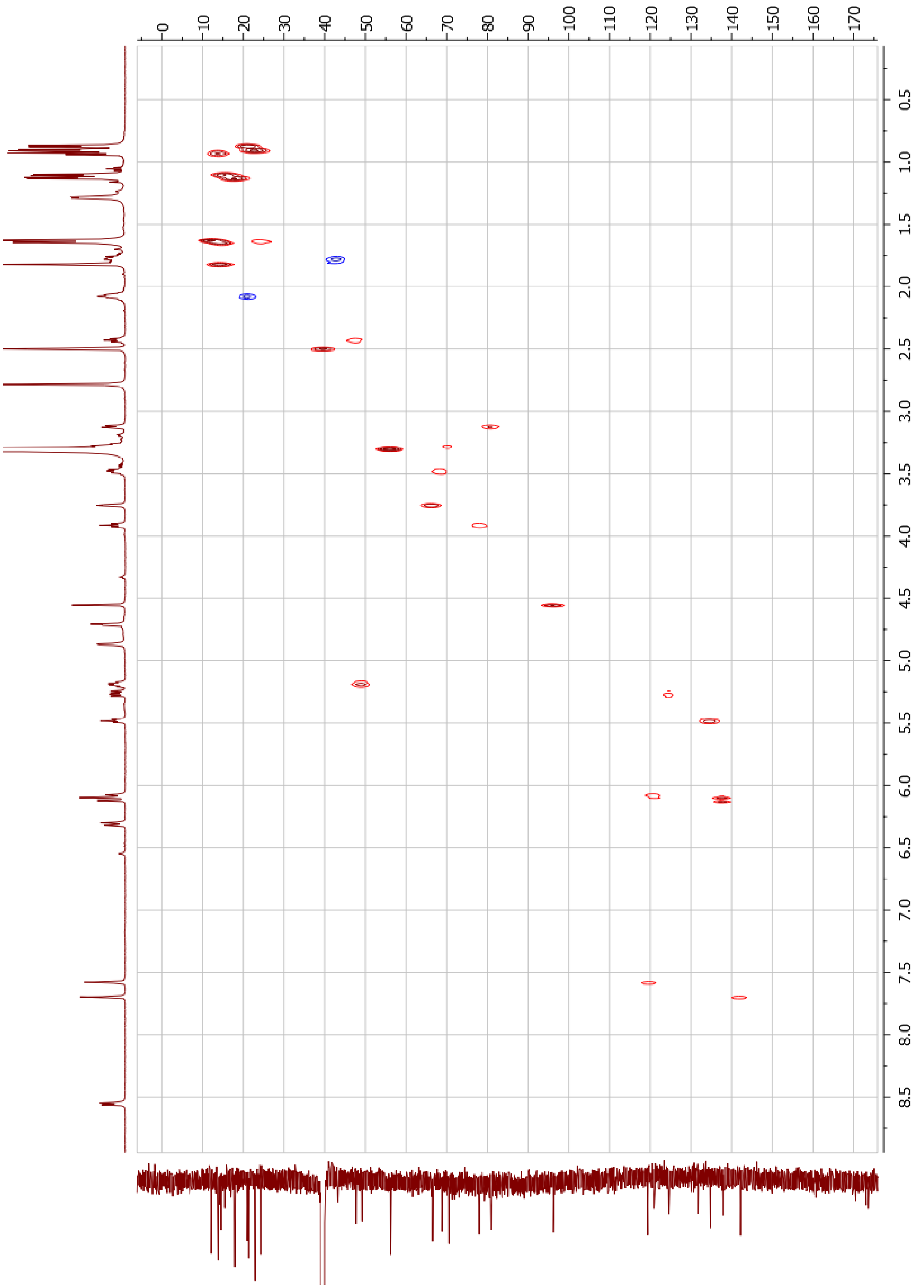

Figure S12. HSQC of iezoside B (**2**) in DMSO-*d*$_6$.

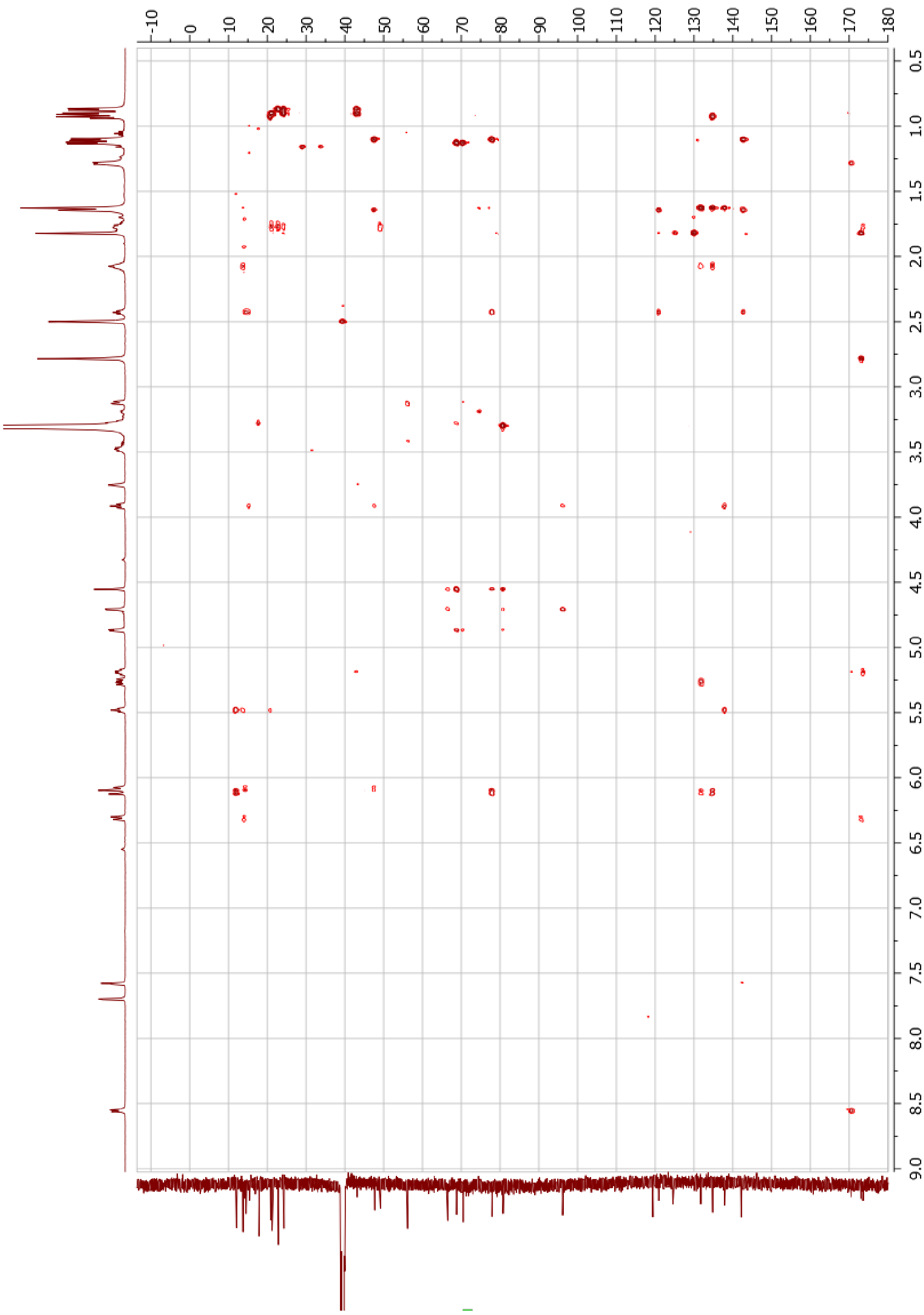

Figure S13. HMBC of iezoside B (**2**) in DMSO-d_6.

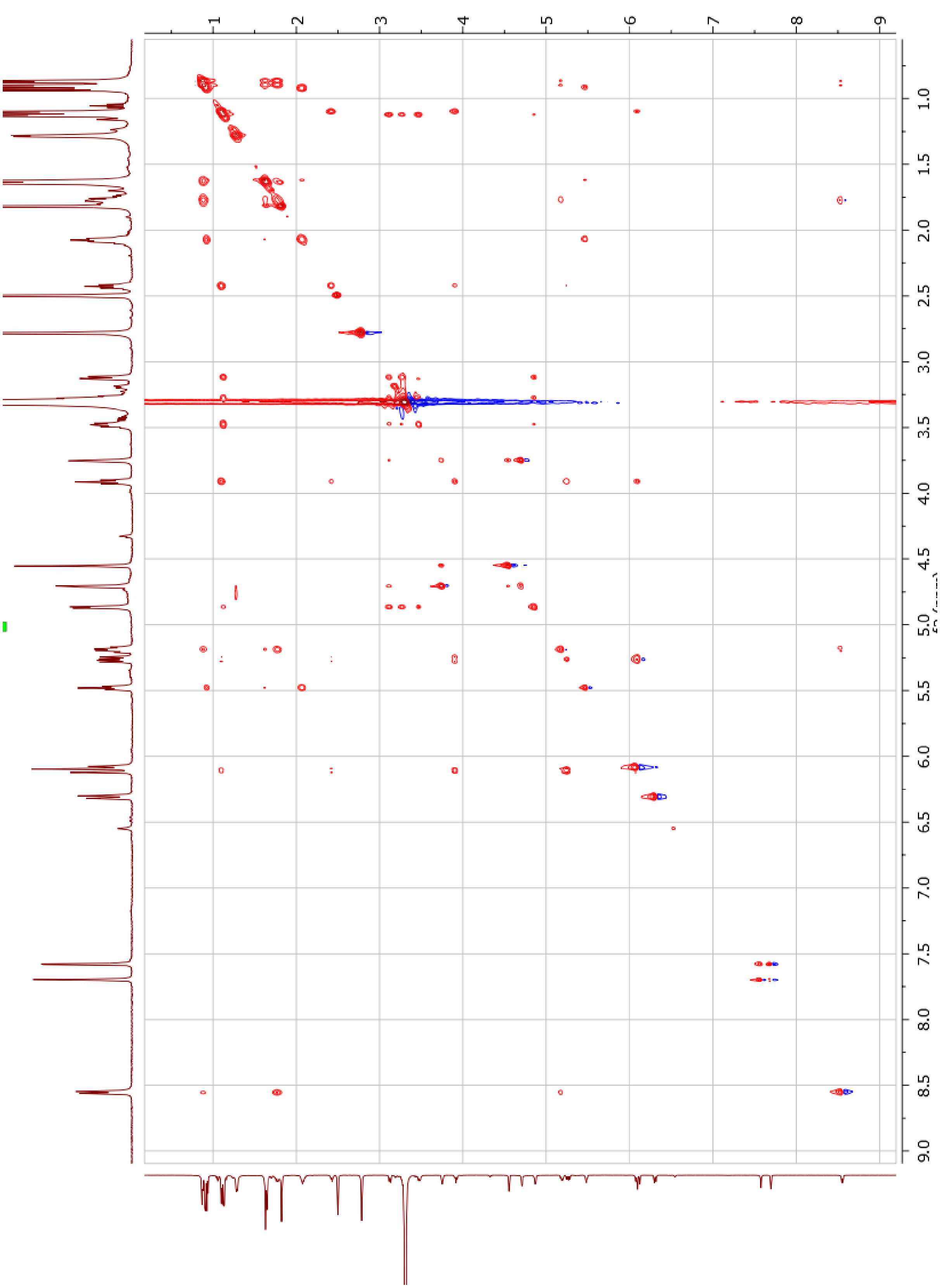

Figure S14. TOCSY of iezoside B (**2**) in DMSO-d_6.

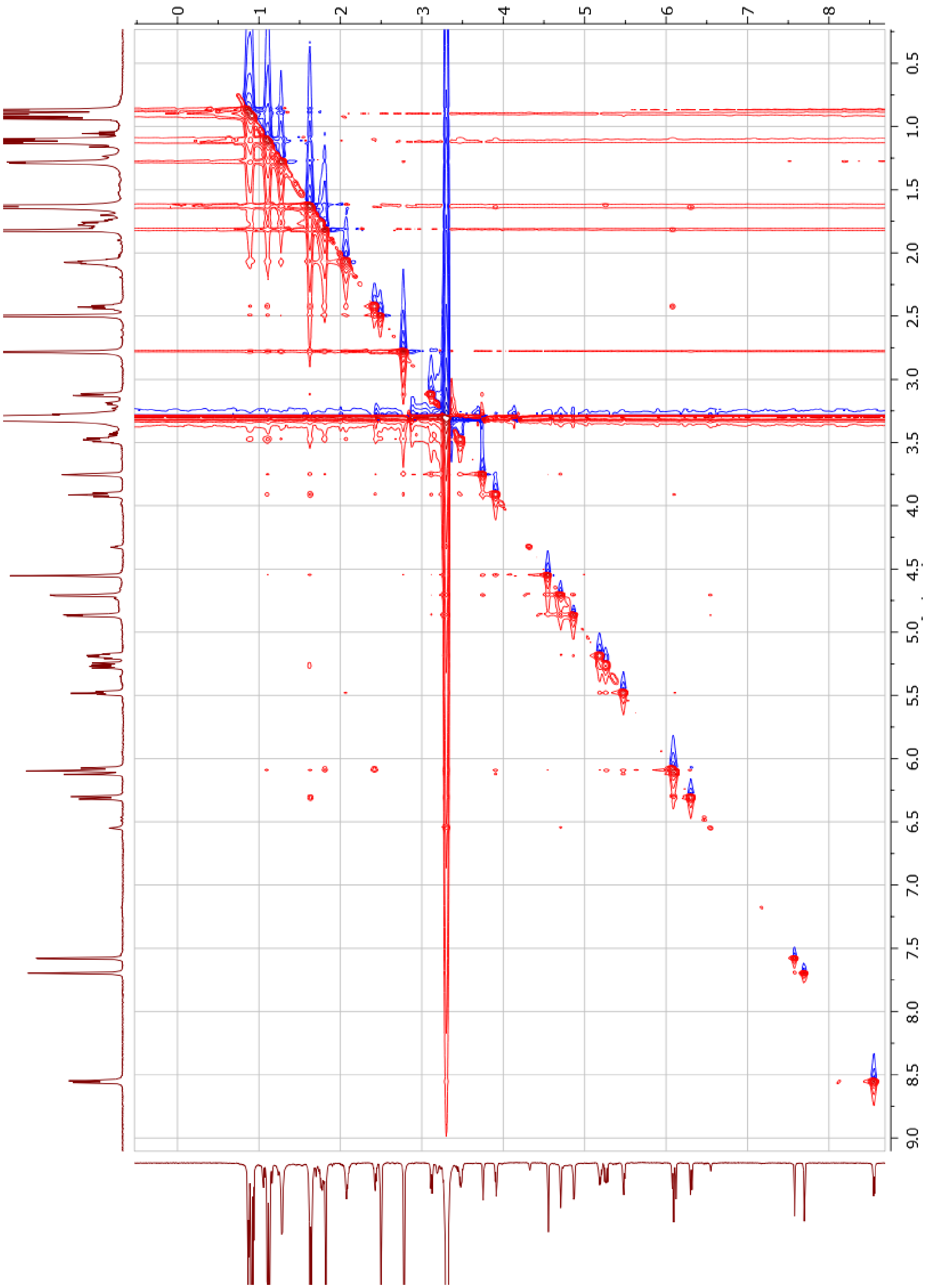

Figure S15. NOESY of iezoside B (**2**) in DMSO-d_6.

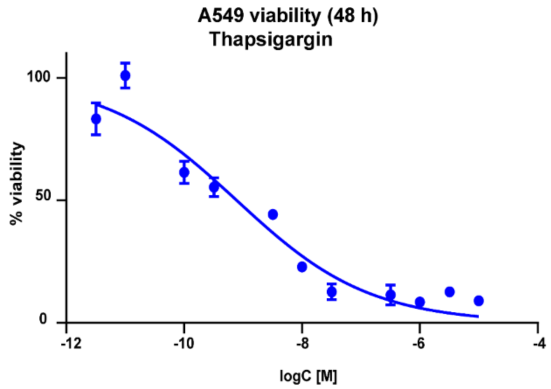

Figure S16. Dose-response curve (cell viability) for thapsigargin in A549 cells at 48 h.

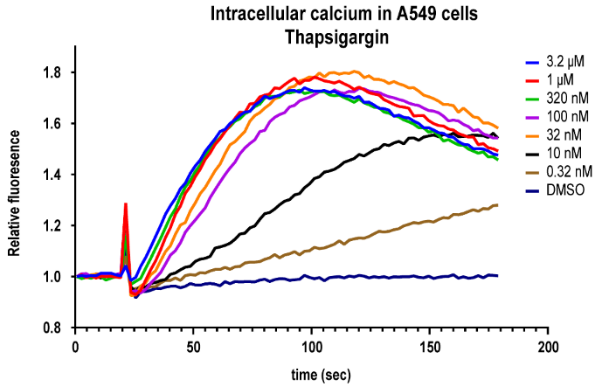

Figure S17. Effect of thapsigargin on cytosolic calcium in A549 cells.

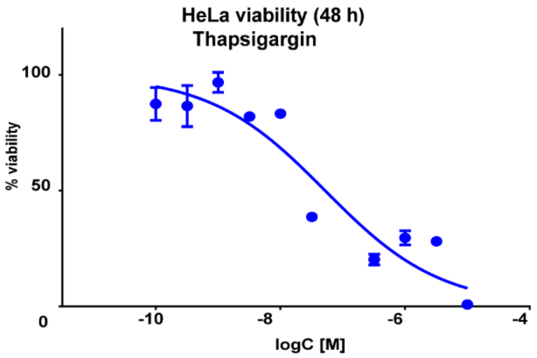

Figure S18. Dose-response curve (cell viability) for thapsigargin in HeLa cells at 48 h.

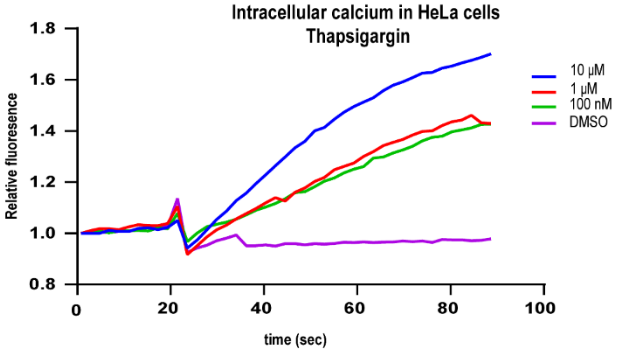

Figure S19. Effect of thapsigargin on cytosolic calcium in HeLa cells.

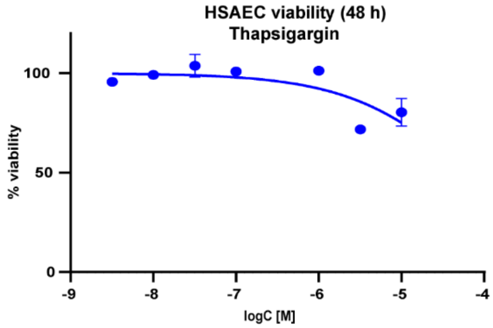

Figure S20. Dose-response curve (cell viability) for thapsigargin in HSAEC cells at 48 h.

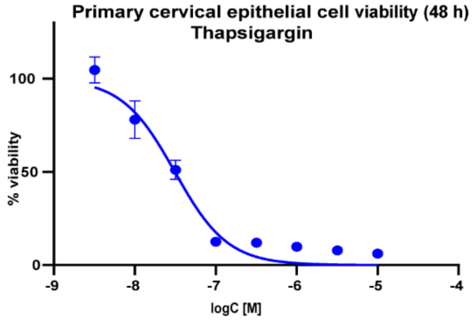

Figure S21. Dose-response curve (cell viability) for thapsigargin in primary cervical epithelial cells at 48 h.

Reference

(1) Kurisawa, N.; Iwasaki, A.; Teranuma, K.; Dan, S.; Toyoshima, C.; Hashimoto, M.; Suenaga, K. Structural Determination, Total Synthesis, and Biological Activity of Iezoside, a Highly Potent Ca^{2+}-ATPase Inhibitor from the Marine Cyanobacterium *Leptochromothrix valapuliae*. *J. Am. Chem. Soc.* **2022**, *144*, 11019–11032. https://doi.org/10.1021/jacs.2c04459.
